# eIF4G1 N-terminal intrinsically disordered domain is a multi-docking station for RNA, Pab1, Pub1, and self-assembly

**DOI:** 10.3389/fmolb.2022.986121

**Published:** 2022-09-23

**Authors:** Belén Chaves-Arquero, Santiago Martínez-Lumbreras, Nathalie Sibille, Sergio Camero, Pau Bernadó, M. Ángeles Jiménez, Silvia Zorrilla, José Manuel Pérez-Cañadillas

**Affiliations:** ^1^ Department of Biological Physical Chemistry, Institute of Physical-Chemistry “Rocasolano”, CSIC, Madrid, Spain; ^2^ Centre de Biochimie Structurale (CBS), CNRS, INSERM, Univ. Montpellier, Montpellier, France; ^3^ Department of Cellular and Molecular Biology, Biological Research Center, CSIC, Madrid, Spain

**Keywords:** intrinsically disordered proteins (IDP), eIF4G1, Pab1, Pub1, NMR, structural ensemble, paramagnetic relaxation enhancement (PRE), SAXS (small-angle X-ray scattering)

## Abstract

Yeast eIF4G1 interacts with RNA binding proteins (RBPs) like Pab1 and Pub1 affecting its function in translation initiation and stress granules formation. We present an NMR and SAXS study of the N-terminal intrinsically disordered region of eIF4G1 (residues 1–249) and its interactions with Pub1, Pab1 and RNA. The conformational ensemble of eIF4G1_1-249_ shows an α-helix within the BOX3 conserved element and a dynamic network of fuzzy π-π and π-cation interactions involving arginine and aromatic residues. The Pab1 RRM2 domain interacts with eIF4G1 BOX3, the canonical interaction site, but also with BOX2, a conserved element of unknown function to date. The RNA1 region interacts with RNA through a new RNA interaction motif and with the Pub1 RRM3 domain. This later also interacts with eIF4G1 BOX1 modulating its intrinsic self-assembly properties. The description of the biomolecular interactions involving eIF4G1 to the residue detail increases our knowledge about biological processes involving this key translation initiation factor.

## Introduction

The eukaryotic translation initiation factor eIF4G is a central player in the regulation of protein expression. First, it enhances translation initiation, the rate-limiting step, by mRNA 3′/5′-end circularization (Tarun et al., 1997; Wells et al., 1998; Preiss and Hentze, 1999; Sonenberg and Hinnebusch, 2009; Aitken and Lorsch, 2012). Structurally this is achieved by the formation of a “closed-loop” complex (CLC), in which eIF4G forms a stable eIF4F heterotrimer (eIF4G + eIF4E + eIF4A) that recognizes the 5′-cap (via eIF4E) of the mRNA and recruits the 3′ poly(A) tail-associated Pab1. On the other hand, eIF4G is involved in the nucleation of stress granule (SG), membrane-less organelles that store components of the translation machineries in an arrested state in response to nutrient starvation, temperature, oxidative or chemical stresses (Kedersha et al., 1999; Hoyle et al., 2007; Buchan et al., 2008, 2011; Jain et al., 2016).

There are two eIF4G genes in *Saccharomyces cerevisiae* that have similar domain architecture (Goyer et al., 1993). EIF4G1 (also referred to as Tif4631) is the most abundant and contains up to 57.4% of its residues in predicted intrinsically disordered regions (IDRs) (MobiDB: https://mobidb.bio.unipd.it/P39935): in the N-terminus (residues 1–77; 111–392; 398–409), middle (residues 481–591) and C-terminus (residues 870–952). The remaining regions contains the domains for interaction with eIF4A (Schütz et al., 2008) and eIF4E (Hershey et al., 1999) that form the eIF4F heterotrimer. EIF4G1 has three RNA binding domains (RNA1, RNA2, and RNA3) within the IDRs (Berset et al., 2003; Park et al., 2011), and a conserved box (BOX3, in the N-terminus IDR) that interacts with Pab1 (Tarun and Sachs, 1996; Kessler and Sachs, 1998) to promote the assembly of the CLC and therefore translation initiation. Conversely, the IDRs also contain the binding sites of translational represors such as Pub1 (Santiveri et al., 2011) (at the N-terminal IDR); Sbp1 (middle IDR), Scd6 (C-terminal IDR) and Npl3 (middle and C-term IDRs) (Poornima et al., 2016), and Ded1 (C-terminal IDR) (Hilliker et al., 2011).

All of these eIF4G-interacting proteins are RNA binding proteins as well and contain a combination of folded domains and IDRs. Pab1 and Pub1, those interacting at the eIF4G1 N-terminal IDR, contain four and three RNA Recognition Motifs (RRM), arranged as bead-on-string, and various low complexity domains (LCD). These proteins are constituents of biological condensates and, in response to temperature increase or acidic pH, undergo *in vitro* LLPS in which both the RRMs and the LCD participate (Lin et al., 2015; Riback et al., 2017; Kroschwald et al., 2018). Pab1, Pub1, and eIF4G1 are the protein constituents of the EGP-bodies, the earliest type of SG described in yeast (Hoyle et al., 2007).

Knowing the structural details of the protein/RNA network involving eIF4G1, Pab1, and Pub1 will help to propose integrative models of translation regulation that include activation and repression pathways. However, because these interactions involve IDRs their study is difficult with X-ray crystallography or Cryo-EM. Therefore, we performed an NMR/SAXS structural study of the eIF4G1 N-terminal IDR and generate an “all-atoms” ensemble stabilized by cation-π and π-π transient contacts. Furthermore, we mapped the binding sites of Pab1, Pub1, and RNA, and show the simultaneous binding of the two RNA Binding Proteins (RBP), reconfigure the conformation of eIF4G1 IDR inducing self-assembly through its conserved element BOX1. The implications of these findings for translation regulation and biomolecular condensation are discussed.

## Results

### The N-terminal eIF4G1 IDR contains residual structural features

**FIGURE 1:**
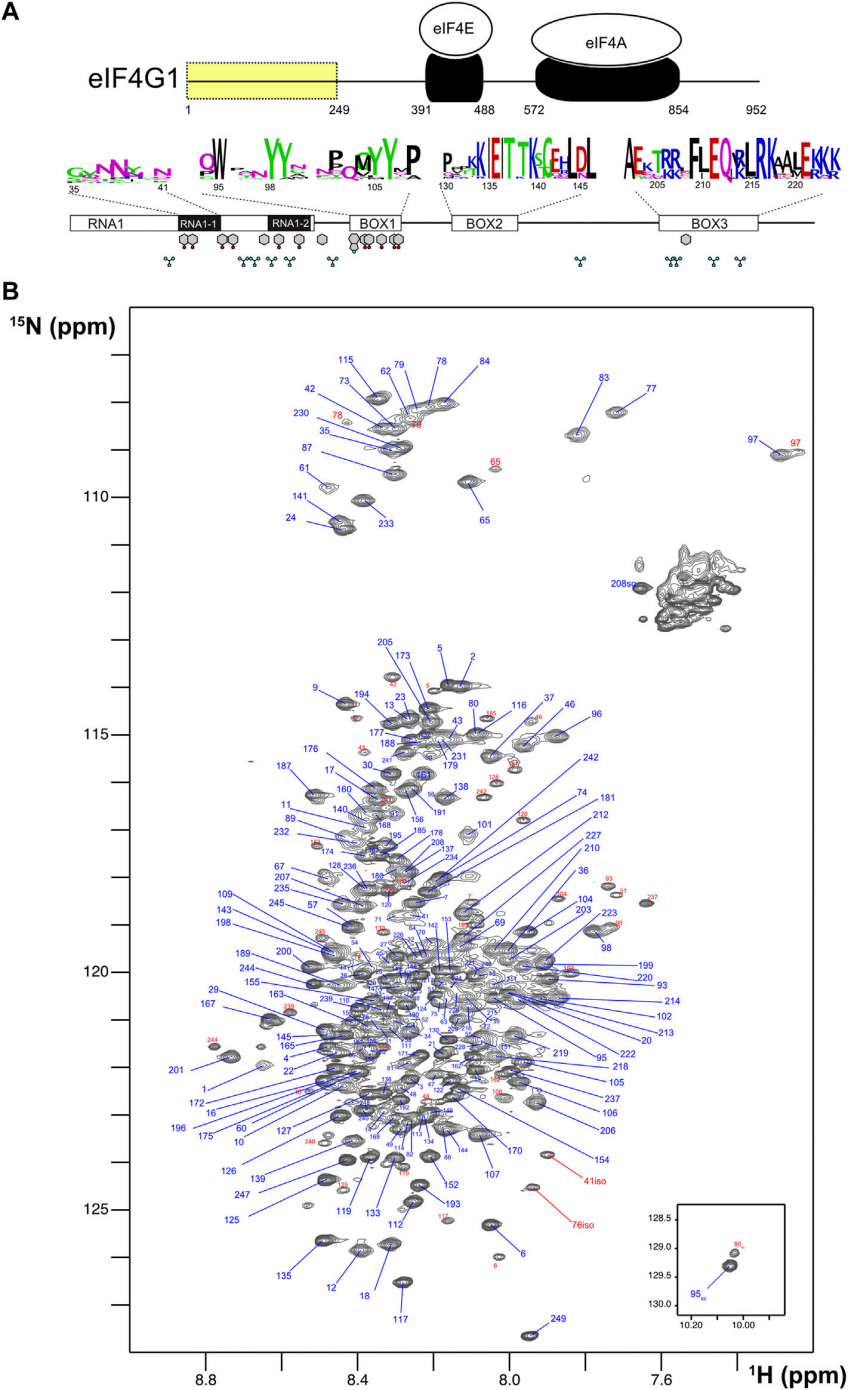
Sequence features and NMR spectrum of eIF4G1_1-249_. **(A)** Schematic representation of *S. cerevisiae* eIF4G1 showing the interaction domains for the other two components of the eIF4F heterotrimer (in black). The N-terminal region containing the conserved boxes (residues 1–249) is highlighted in yellow. The conserved features (BOXes) of eIF4G1_1-249_ are represented below including weblogos indicating the conservation of each box across *Saccharomyces* and the position of key side chains (Tyr, Phe, Trp, and Arg) capable of π-π and π-cation interactions. **(B)** ^1^H-^15^N HQSC spectrum of eIF4G1_1-249._ Each assigned residue is labeled: blue, major form; red, minor species.

The N-terminal IDR of *Saccharomyces cerevisiae* eIF4G1 contains three boxes of about 15–20 conserved residues each, and a conserved RNA binding region (RNA1), within the first 249 amino acids (Park et al., 2011) (Figure 1A). We studied by NMR the conformational properties of eIF4G1_1-249_, a construct that is stable for days under different pH conditions (Supplementary Figure S1). NMR is a very powerful technique for the investigation of IDRs and their interactions at the residue level [(Kurzbach et al., 2015; Gibbs et al., 2017; Milles et al., 2018) and references therein].

The ^1^H-^15^N HSQC NMR spectrum of eIF4G1_1-249_ is characteristic of an intrinsically disordered protein (IDP) with low dispersion in the proton dimension and sharp signals (Figure 1B). However, interestingly, several glycines showed relatively broad peaks at *δ*_NH_ < 8.00 ppm (e.g., G97, G77, and G83) that are not compatible with a fully disordered state. We identified several minor species (signals labelled in red) that correspond to *cis*Pro conformers, and two uncommon chemical isomerization forms at positions 41 and 76 that were assigned to isoaspartates (Supplementary Figure S2). These variants arise from deamidation of N41 and N76, that lie next to Gly residues in the protein sequence; Asn-Gly sequences are known to have the highest tendency to experience this non-enzymatic deamidation in model peptides (Robinson and Robinson, 2001). The level of deamidation is similar for the two positions (12%–14%) and remained constant in different samples and over NMR experimental time, suggesting that these forms might have been generated *in vivo*.

**FIGURE 2:**
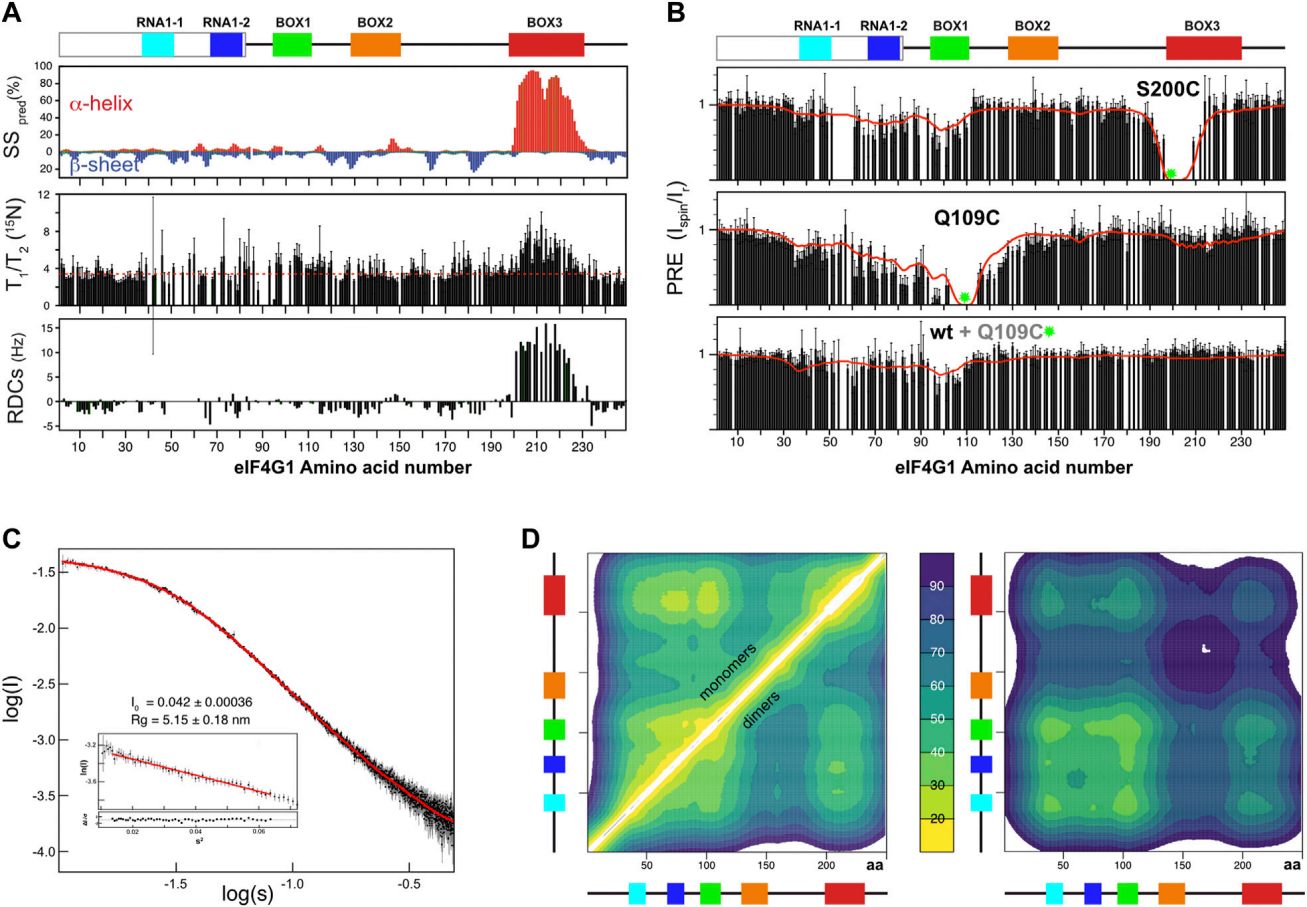
NMR structural analysis of eIF4G1_1-249_. **(A)** NMR evidence of residual secondary structure (per residue): percentage of predicted secondary structure (SS pred) calculated using the d2D program (Camilloni et al., 2012) (upper graph), ^15^N relaxation T_1_/T_2_ (middle graph, dotted red line marks the average) and residual dipolar couplings (RDCs) (lower graph). Conserved sequence elements in eIF4G1_1-249_ are represented at the top of the figure. **(B)** Per residue effect of paramagnetic relaxation enhancement (PRE) over relative signal intensities of two individual ^15^N-labelled eIF4G1_1-249_ mutants and of ^15^N-labelled wild-type eIF4G1_1-249_ mixed (1:1) with spin-labeled eIF4G1_1-249_ Q109C at natural isotopic abundance. Green circles mark the position of spin-labels. The red lines indicate the back-calculated PRE effects across the 500-member ensembles of monomers (upper and middle graphs) and dimers (lower graph). **(C)** Experimental SAXS curve showing log of intensity (I) versus log of scatter (S) of eIF4G1_1-249_ and EOM fitting obtained from the eIF4G1_1-249_ atomic models (red curve). Inset: Guinier analysis and the derived Radius of gyration (R_g_) and forward scattering intensity [I (0)] values. **(D)** (left panel) Ensemble-averaged intramolecular Cα contact maps obtained from the monomers and dimers (upper and lower triangles, respectively), and (right panel) intermolecular Cα contact maps. The average distances (in Å) were color-coded according to the scale in the middle, and the conserved boxes of eIF4G1_1-249_ are indicated along the axes of each map.

Analysis of eIF4G1_1-249_ secondary structure based on ^13^C chemical shifts, T_1_/T_2_^15^N relaxation times and residual dipolar couplings (RDCs) revealed the presence of an α-helix within BOX3 (Figure 2A). This finding was confirmed by characteristic sequential amide-amide NOEs measured in a 3D ^1^H-^15^N-HSQC-NOESY-^1^H-^15^N HSQC spectrum (Supplementary Figure S3). We determined the NMR structure of this α-helix using a BOX3 model peptide (eIF4G1_187-234_) (Supplementary Figure S3). No further standard secondary structure elements were identified in eIF4G1_1-249_. However, the broad glycine peaks seen in Figure 1B, suggested the presence of residual higher order structures in this construct. In support of such structures, random coil index” (RCI) values S2 predicted from the chemical shifts (Camilloni et al., 2012), and the lower T_1_, T_2_ relaxation times for the conserved boxes suggested that these boxes might be involved in transient contacts that restrict mobility and/or induce chemical exchange processes resulting in short T_2_ values (Supplementary Figure S4). The existence of long-range interactions was evidenced by paramagnetic relaxation enhancement (PRE) measurements. The nitroxyl spin-label derivatization of engineered cysteine mutants (eIF4G1_1-249_ has no native Cys) indicates long-range PREs for S200C and Q109C mutants (Figure 2B). In a protein such as eIF4G1_1-249,_ the PREs [calculated as described in (Battiste and Wagner, 2000) and Supplementary Figure S5] are expected to occur within 25–30 Å of the spin label. Therefore, the PRE data suggested the presence of long-range contacts in eIF4G1_1-249_ involving BOX1, RNA1, and BOX3.

To determine if these contacts are predominantly intra- or intermolecular, we placed the spin label in the non-isotope labeled Q109C mutant, added wild-type ^15^N-labeled eIF4G1_1-249_, and then measured PRE. The PRE fingerprint shows that eIF4G1_1-249_ can self-interact through contacts involving BOX1 and RNA1, as these elements “sense” the presence of the spin label in *trans* (Figure 2B lowest graph). However, the magnitude of the effects of the spin label in *trans* is smaller than when it is in *cis* (Figure 2B middle graph), suggesting that there is a small population of the self-associated species. All of the Tyr residues of the construct are contained in these regions involved in eIF4G1_1-249_ self-recognition (see Figure 1A). Five of these Tyr resides are included in BOX1 that is a predicted amyloid-like sequence (Supplementary Figure S6). These data suggest that eIF4G1_1-249_ self-recognition involves Tyr-Tyr interactions.

### eIF4G1 IDR conformational ensemble

IDPs, are considered to consist of ensembles of co-existing conformers. To build realistic conformational ensembles of IDPs, it is necessary to identify the possible residual secondary structures and long-range interactions between different regions of the polypeptide (Schwalbe et al., 2014). These structural features are generally sparsely populated and transient in IDPs, which makes them difficult to detect and quantify experimentally thereby undermining the possibilities of calculating conformational ensembles. Nevertheless, several studies have proposed that interactions that favor aggregation, flexibility and/or long-range contacts are prevalent in IDPs (Brangwynne et al., 2015; Vernon et al., 2018; Gomes and Shorter, 2019). In particular on the key role of cation-π and π-π interactions in the “molecular grammar” of phase separation in prion-like IDPs (Wang et al., 2018). There are 11 Arg, 11 Tyr, 3 Phe, and 1 Trp in eIF4G1_1-249_ that are suitable for these types of interactions, and they are mostly located in the conserved boxes (see Figure 1A). Therefore, we hypothesized that cation-π and π-π interactions (involving Arg, Tyr, Phe, and Trp) might dominate the long-range contacts in eIF4G1_1-249_.

Different methods have been used to calculate IDP conformational ensembles using experimental data (NMR, SAXS and others) and/or computational approaches (Bernadó et al., 2005; Kragelj et al., 2015; Kurzbach et al., 2015; Das et al., 2018; Bhattacharya and Lin, 2019; Estaña et al., 2019). Here we used the algorithm in the program Cyana 3.0 (Güntert and Buchner, 2015) for fast generation of eIF4G1_1-249_ all-atoms structural models. This approach allows a straightforward implementation of: 1) NOE-derived distance restraints and ^13^C-derived φ/ψ dihedral restraints for the parts of the protein that are well-folded (i.e., BOX3) and 2) ambiguous restraints for the cation-π and π-π interactions (involving Arg and Tyr) that we propose as dominant transient residue-residue contacts. We refer to these latter types of restraints as “knowledge-based” and used cautions to avoid biases in their selection (see materials and methods for specific details). We calculated 80,000 eIF4G1_1-249_ structures and sorted them using our own greedy algorithm that optimized the fitting to experimental PRE data stepwise. As a seed for the ensemble the protocol chooses the structure with minimum PRE violations and then continues building up the ensemble stepwise using the same criteria (i.e., incorporating the structure that, together with the previously selected structures(s), minimizes violations). The target function reached a minimum value relatively quickly and increased slowly afterwards (Supplementary Figure S7). We arbitrarily selected a final 500-member ensemble to ensure sufficient structural variability while still maintaining good agreement between the back-calculated and the experimental PREs (red line in Figure 2B). Because the intermolecular PRE data showed self-association, we performed a similar protocol for analysis of eIF4G1_1-249_ dimers, hypothesizing that Tyr-Tyr interactions are the driving force of dimerization. The SAXS curve evidenced the IDP nature of eIF4G1_1-249_ (Figure 2C). To validate the eIF4G1_1-249_ ensemble models, we applied the Ensemble Optimization Method (EOM) genetic algorithm (Bernadó et al., 2007; Tria et al., 2015) to model the SAXS curve. Pools for monomeric and dimeric conformations were used without restricting the relative percentages of each set. We performed 10 independent EOM calculations, and each of them resulted in excellent fittings of the experimental curve (Figure 2C). Importantly, similar calculations done with either eIF4G1_1-249_ monomers or dimers alone resulted in worse fits. In the SAXS-selected eIF4G1_1-249_ ensemble, the monomers dominate (88%). As expected, the back-calculated PREs from the collection of EOM ensembles showed poorer agreement with the experimental data, but neatly reflected the overall trends regarding the long-range contacts in eIF4G1_1-249_ (Supplementary Figure S7).

The eIF4G1_1-249_ ensemble of conformers showed a flexible α-helix in BOX3 and, despite the presence of long-range contacts, no predominant tertiary fold was found. The average Cα-Cα distance maps revealed that local and long-range contacts were prevalent between BOX1 and RNA1-1/RNA1-2 boxes, and between these three elements and BOX3 (Figure 2D left). In contrast, there was a remarkable absence of long-range interactions involving BOX2. Dimerization contacts were dominated by BOX1 and to a lesser extent by the RNA1-1 box (Figure 2D right). The BOX3 region also showed a minimum in the intermolecular Cα-Cα distance maps, due to the coexistence of intramolecular Arg-Tyr and intermolecular Tyr-Tyr contacts (Figure 2D right). Indeed, these π-π and cation-π interactions tend to appear in networks, rather than in binary mode, probably favored by the planar nature of aromatic and guanidinium groups.

In summary, these data showed that eIF4G1_1-249_ is predominantly disordered except for an α-helix in BOX3. Atomistic models were constructed with experimental and knowledge-based restraints and ensembles were built by restraining against experimental NMR and SAXS data. Their analysis showed an intrinsic tendency of eIF4G1_1-249_ to dimerize (oligomerize), in which BOX1 plays the chief role.

### eIF4G1 interacts with Pab1 and Pub1 through multiple binding sites

**FIGURE 3:**
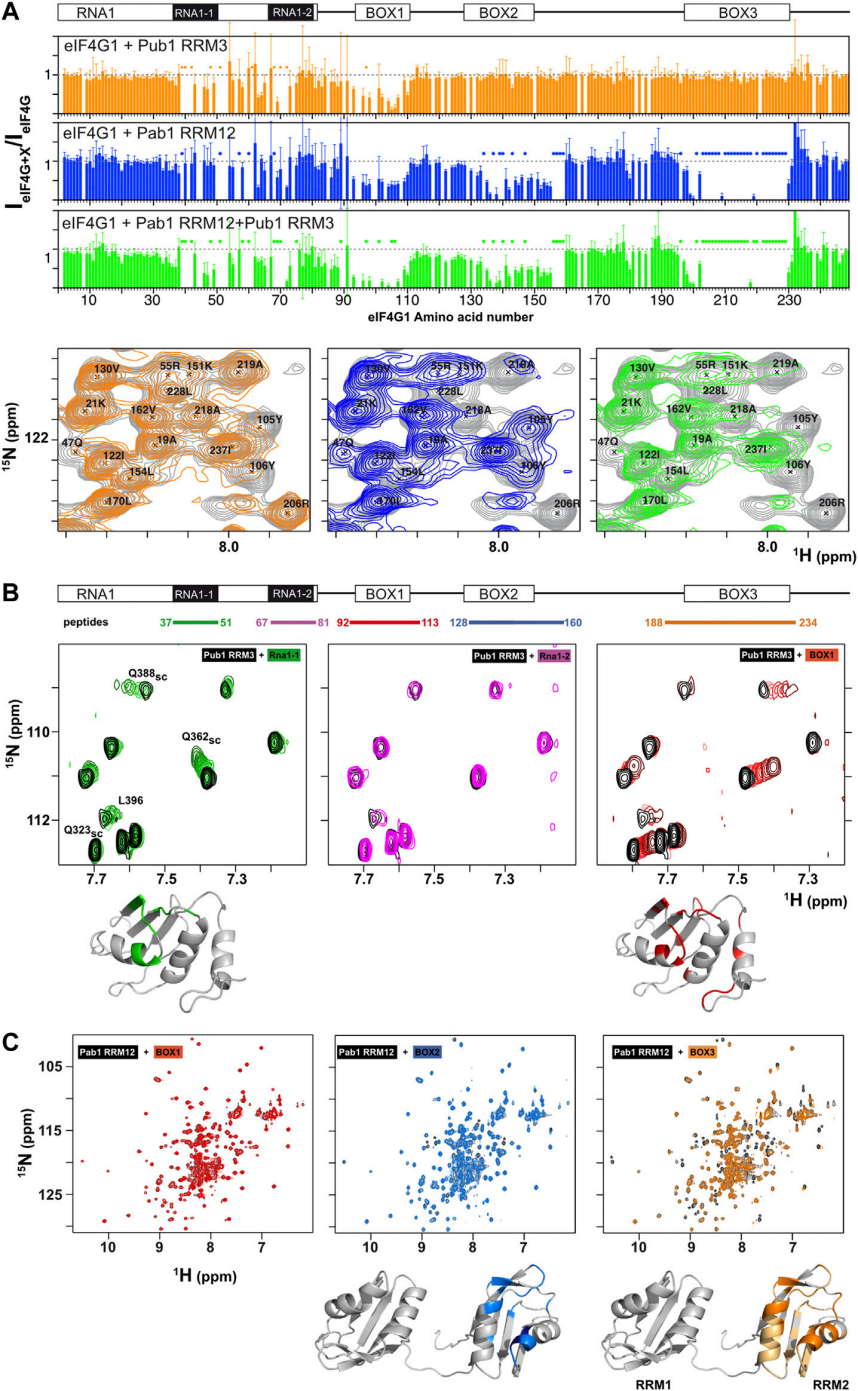
Mapping of Pub1 and Pab1 binding sites on eIF4G1_1-249_. **(A)** Sequence dependence of eIF4G1_1-249_^1^H-^15^N HSQC signal intensity ratios between free eIF4G1_1-249_ and various eIF4G1_1-249_ complexes with Pub1 RRM3 (orange), Pab1 RRM12 (blue) and Pub1 RRM3+Pab1 RRM12 (green). A representative region of the eIF4G1_1-249_^1^H-^15^N HSQC spectrum (in grey) is shown below superimposed with the equivalent spectra of the Pub1 RRM3 (in orange), Pab1 RRM12 (blue) and Pub1 RRM3+Pab1 RRM12 (green) complexes. Specific residues were labelled to illustrate the selective disappearance of eIF4G1_1-249_ signals upon complex formation in each case (marked with dots in the bar charts). **(B,C)** NMR study of the interaction of eIF4G1 peptides with Pub1 RRM3 **(B)** and Pab1 RRM12 **(C)** monitored on their ^1^H-^15^N HSQC spectra. The regions of eIF4G1_1-249_ that correspond to each of the five peptides tested are indicated at the top in **(B)**. Black: spectra of the free proteins; Colors: peptide titrations according to the color scheme in **(B)**. Chemical shift perturbations and signal broadening were mapped onto the model structures of Pub1 RRM3 (PDB:2LA4) and Pab1 RRM12 (bottom).

eIF4G1, Pub1, and Pab1 that are considered as key components of SG (Buchan et al., 2011; Jain et al., 2016) We studied the structural details of their interaction network. Previous to this work, we showed that Pub1 RRM3 interacts with eIF4G1 (Santiveri et al., 2011). Here we mapped this interaction on eIF4G1_1-249_ by analysis of changes on the ^1^H-^15^N HSQC spectra**,** which indicated three putative binding sites for RRM3 in eIF4G1_1-249_ (orange bar chart in Figure 3A): two in RNA1 (RNA1-1 and RNA1-2) and one in BOX1. Strikingly, these sites have a small consensus sequence motif (YNNxxxY), only present in this region of the eIF4G1. We tested the ability of short peptides of eIF4G1 that corresponded to the conserved elements (Figure 3B) to bind to ^15^N-labelled Pub1 RRM3, by monitoring their effect on the Pub1 RRM3 ^1^H-^15^N HSQC spectrum. Only BOX1 and RNA1-1 peptides caused changes in the Pub1 spectrum arising from direct contacts (Figure 3B). The Pub1 binding site in BOX1 overlaps with the amyloid-like sequence (Supplementary Figure S6). The absence of spectral changes upon RNA1-2 peptide titration (Figure 3B) suggested that the effects observed on the spectrum of the corresponding region of eIF4G1_1-249_ in complex with Pub1 RRM3 (orange bar chart in Figure 3A) are probably due to conformational rearrangements.

**FIGURE 4:**
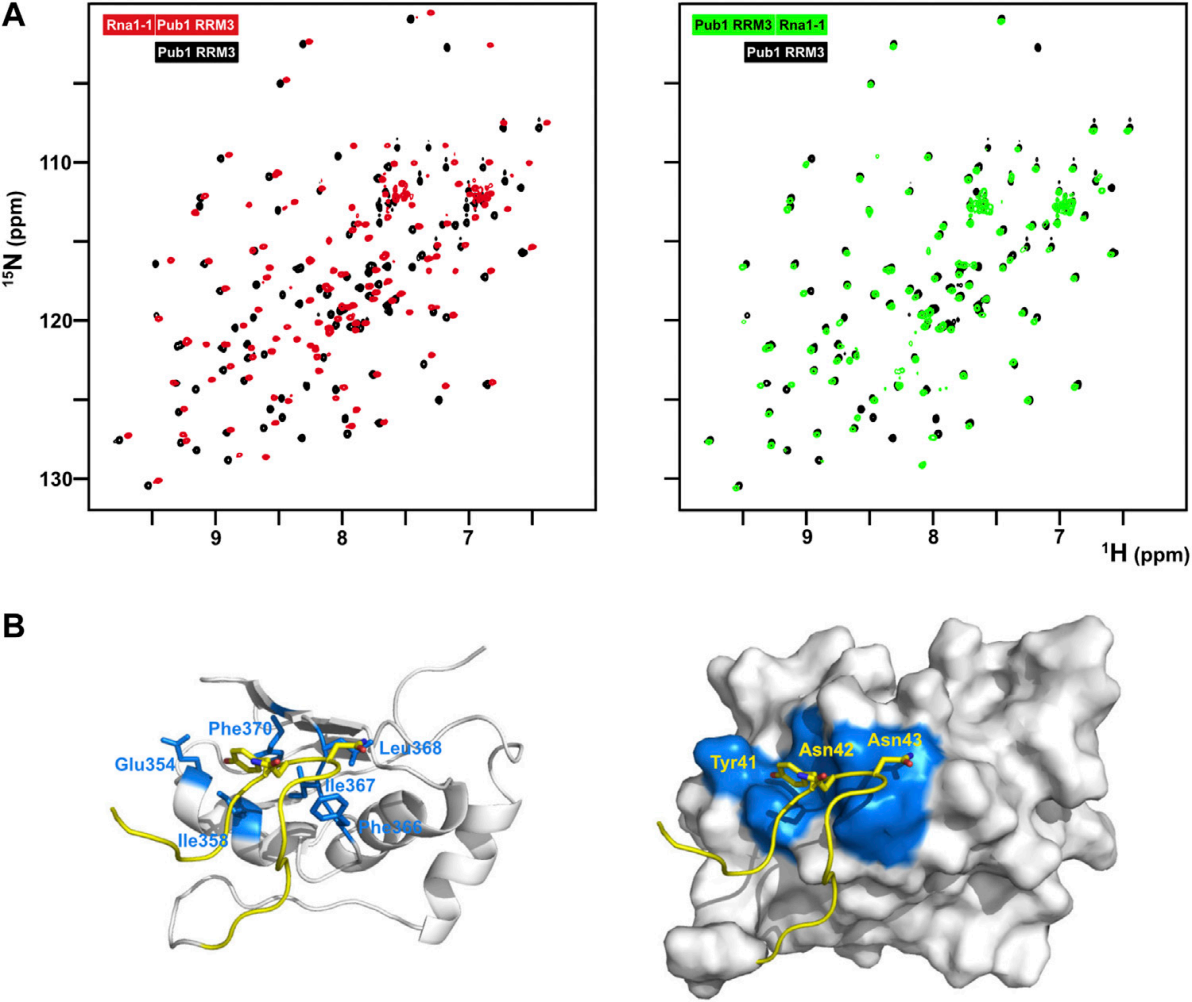
Structural characterization of the eIF4G1-Pub1 interaction. **(A)** Superposition of 2D ^1^H-^15^N HSQC of free Pub1 RRM3 (black) on either eIF4G1_35-49_-Pub1 RRM3 (red) or Pub1 RRM3-eIF4G1_35-49_ (green) chimeric constructs. **(B)** NMR structure of eIF4G1_35-49_-Pub1 RRM3. Yellow, eIF4G1; White, Pub1 RRM3; Blue, Pub1 RRM3 residues contacting eIF4G1. Key interacting residues are labelled on both eIF4G1 and Pub1RRM3 regions of the chimera.

The binding of short eIF4G1 peptides was too weak to obtain structural restraints (e.g., intermolecular NOEs) for calculation of the structure of the complex. To overcome this technical problem, we constructed recombinant chimeras of eIF4G1_35-49_ and Pub1 RRM3 (Figure 4; Supplementary Figure S8). The NMR spectrum of the eIF4G1 peptide fused to the C-terminus of Pub1 was similar to that of Pub1 RRM3 alone (Figure 4A right), whereas that of the N-terminally fused chimera differed significantly (Figure 4A, left), suggesting that the peptide can effectively fold-back into the binding site only in the latter case. Using this latter construct, we obtained enough experimental restraints to calculate the structure of the eIF4G1_35-49_-Pub1 RRM3 chimera (PDB: 6Z29), which shed light on the key elements required for molecular recognition (Figure 4B and Supplementary Figure S8). The structure showed that eIF4G1 residues Y_41_, N_42_ and N_43_ (part of the YNNxxxY conserved motif) interact with a shallow cleft in Pub1 RRM3, defined by the contact between helix α1 and strand β2. The eIF4G1 Y_41_ is inserted into a small cavity and contacts I_358_, I_367_ and F_370_ of Pub1, whereas the N_42_ and N_43_ of eIF4G1 are more exposed but contact L_368_ and F_366_ of Pub1. The latter residue was previously shown to be important for eIF4G1-Pub1 interaction (Santiveri et al., 2011). The interaction surface was small, in agreement with a weak eIF4G1-Pub1 interaction.

We next studied the interaction between eIF4G1 and Pab1 using similar approaches. NMR titrations of unlabeled Pab1 RRM12 over ^15^N-eIF4G1_1-249_ (blue bar chart in Figure 3A) caused similar pattern of perturbations and signal disappearance than Pub1 RRM3 but with additional changes in BOX2 (aa 135–160) and BOX3 (aa 200–234). Experiments with eIF4G1_1-82_ shows little changes for Pab1 RRM12 titration in comparison with Pub1 RRM3 (Supplementary Figure S9), suggesting that Pab1 RRM12 does not interact with RNA1 and changes could be due to conformational reorganization of eIF4G1_1-249_. For the other elements, only BOX3 has been reported as a Pab1 binding site to date (Kessler and Sachs, 1998). As done for Pub1 RRM3, we studied the interactions of Pab1 RRM12 with elF4G1 fragments to validate the putative binding sites. As expected, the BOX3 peptide interacted with Pab1 RRM12 causing significant perturbations in the helix1-helix2 interface of RRM2 (Figure 3C right), an equivalent region to that involved in human eIF4G-PABP1 recognition (Safaee et al., 2012). The BOX2 peptide also interacted with RRM2 through a similar interface; but probably weakly because it causes fewer changes than the BOX3 peptide. NMR data showed that the BOX1 peptide does not directly interact with Pab1 RRM12 and that the observed changes in that region of eIF4G1_1-249_ (blue bar chart in Figure 3A) are due to reorganization of internal contacts. Interestingly Pub1 and Pab1 used different interfaces of the RRM to interact with eIF4G1 (Supplementary Figure S8C).

These results showed that, although Pub1 and Pab1 interact with eIF4G1_1-249_ through multiple sites, most of these interactions are weak because they caused minor chemical shift changes in these RBPs; the exception is the Pab1-BOX3 interaction that showed changes of a larger magnitude. The presence of two eIF4G1 binding sites for Pub1 and Pab1, combined with their self-association properties, might suggest a possible cooperative recognition mode of eIF4G1.

### Pub1 and Pab1 induce BOX1-dependent aggregation

**FIGURE 5:**
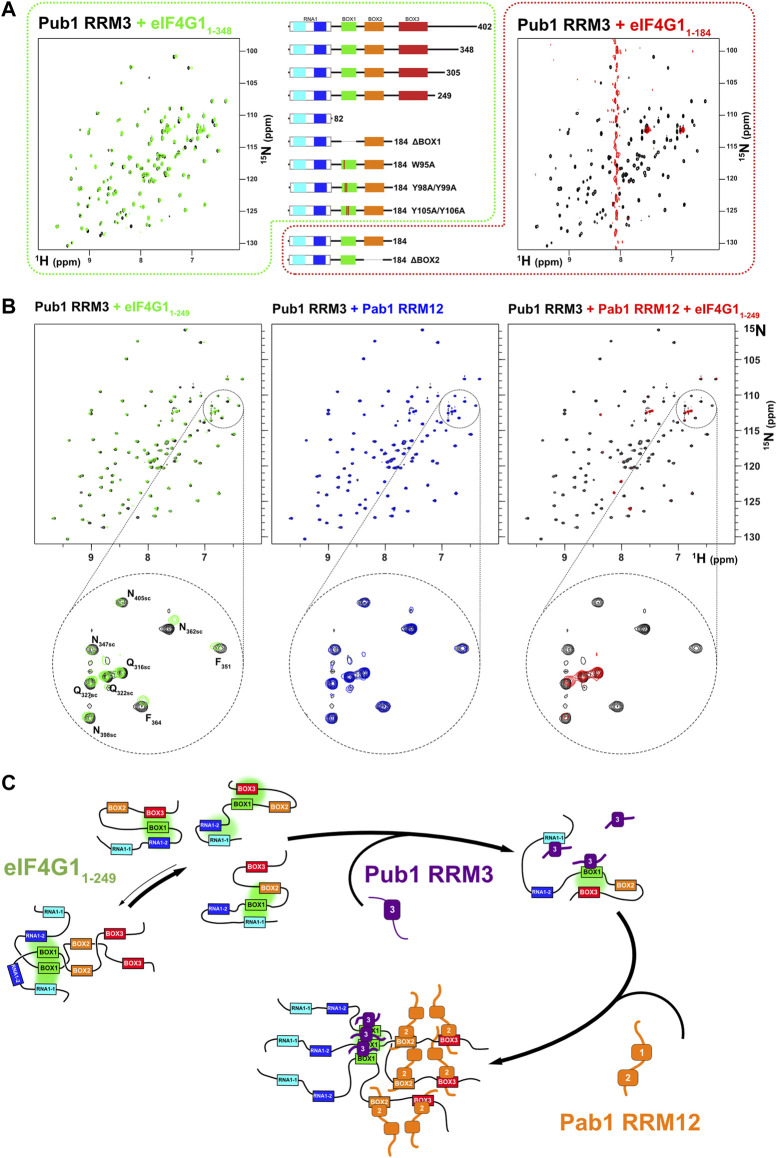
Pub1 RRM3 can interact with eIF4G1 through two different modes. **(A)** Superposition of the ^1^H-^15^N HSQC spectra of free Pub1 RRM3 (black) and Pub1 RRM3 in complex with eIF4G1_1-348_ (green) or eIF4G1_1-184_ (red). The Pub1 RRM3 NMR crosspeaks show small perturbations in the first complex (left panel) and completely disappear (with the exception of the highly mobile Asn/Gln side chain peaks) in the spectrum of the complex (right panel). The central panel shows eIF4G1 mutants that had similar effects as eIF4G1_1-348_ (outlined in green dash) or eIF4G1_1-184_ (outlined in red dash) on the Pub1 RRM3 spectrum. **(B)** Study of the effect of eIF4G1_1-249_ and/or Pab1 RRM12 titrations on the Pub1 RRM3 ^1^H-^15^N HSQC spectrum. Superposition of the Pub1 RRM3 spectra before (black signals) and after titration with eIF4G1_1-249_ (left, green signals), Pab1 RRM12 (middle, blue signals) and eIF4G1_1-249_ + Pab1 RRM12 (right, red signals). A small area of each spectrum is expanded below for a more detailed view. The Pub1 RRM3 signals are unperturbed upon titration with Pab1 RRM12 (blue spectrum), but disappear when Pab1 RRM12 is combined with eIF4G1_1-249_ (red spectrum). **(C)** A dual key interaction model between eIF4G1_1-249_, Pub1 RRM3 and Pab1 RRM12 to explain oligomerization. Multiple intramolecular interactions between BOX1 and other conserved elements of eIF4G1_1-249_ maintain it predominantly monomeric. Weak interactions with Pub1 RRM3 (first key) cancel out some of these transient contacts, but some remain (e.g., BOX1-BOX3) preventing BOX1-driven oligomerization. The interaction with Pab1 RRM12 (second key) further blocks internal contacts to BOX1 triggering its aggregation. Pub1 RRM3 binding to BOX1 oligomeric forms would be reinforced by Pub1-Pub1 interactions.

During the course of the study, we found that Pub1 RRM3 interacts differently with different eIF4G1 constructs. Titration with eIF4G1_1-82_, eIF4G1_1-305_, eIF4G1_1-348,_ and eIF4G1_1-402_ constructs caused small changes in the Pub1 RRM3 NMR signals equivalent to those described for its interaction with eIF4G1_1-249_ and BOX1 and RNA1-1 peptides (Figure 5A left). However, surprisingly, titration with eIF4G1_1-184_ cause the disappearance of nearly all of the Pub1 RRM3 ^1^H-^15^N HSQC crosspeaks (Figure 5A right). This result cannot be explained by changes in the chemical exchange kinetics because signals not affected by binding (i.e., Δω = 0) will not experience line broadening. The massive line broadening is either compatible with Pub1 RRM3 being part of a high molecular weight structure (i.e., aggregates), or it is in equilibrium with these large particles in a way that properties associated to their slow tumbling (T_1_, T_2_) are transferred to free Pub1 RRM3.

We further investigate the cause of Pub1 RRM3 behavior and discovered that it recover the weak-binding pattern with several eIF4G1_1-184_ BOX1 mutants (ΔBOX1, W95A, Y98A/Y99A, and Y105A/Y106A), whereas deletion of BOX2 (ΔBOX2) still caused the Pub1 aggregation-like pattern. Thus, BOX1 is the element causing the differential binding mode of Pub1 RRM3, but how. Our hypothesis is that Pub1 RRM3 binding causes conformational rearrangements on eIF4G1_1-184_ exposing aggregation-prone BOX1 (Supplementary Figure S5A,B). Interestingly, eIF4G1_1-184_ experiences a complex degradation (Supplementary Figure S1) that we characterized by NMR (Supplementary Figure S10). New C-terminal peaks arise from internal breaks at specific points of the polypeptide chain and the signals from the BOX1 region disappear (e.g., W95 sidechain and G97), a behavior compatible with aggregation. Macroscopically, eIF4G1_1-184_ (and eIF4G1_1-184_ ΔBOX1) samples age to hydrogels that bind Congo-red (Supplementary Figure S5C), a dye used to detect amyloids and protein aggregates (Yakupova et al., 2019), but eIF4G1_1-184_ ΔBOX1 does not. As in the case of Pub1 RRM3, these observations suggests that the loss of BOX1 transient interactions to other parts of eIF4G1 (in this case by degradation) trigger its aggregation-prone properties.

The construct eIF4G1_1-249_ is more stable (Supplementary Figure S1) and Pub1 RRM3 binds to it weakly (Figure 5A left). This suggests that Pub1 RRM3 is unable to disrupt the BOX3-BOX1 contacts present in longer eIF4G1 forms (eIF4G1_1-249,_ eIF4G1_1-305_, eIF4G1_1-348_ and eIF4G1_1-402_). Consistent with this hypothesis, titration of Pab1 RRM12 into the eIF4G1_1-249_/Pub1 RRM3 complex retrieve the Pub1 RRM3 broad spectrum, suggesting that the Pab1 RRM12 binding to BOX2 and BOX3 acts as a second switch that releases the BOX1 oligomers. NMR data showed that Pub1 RRM3 did not interact with Pab1 RRM12 (Figure 5B middle panel). These data agree with the data described above in Figure 3A, where the presence of the Pab1+Pub1 mixture (Figure 3A, in green), caused larger BOX1 line broadening in the eIF4G1_1-249_ spectrum than the presence of either Pub1 or Pab1 alone (Figure 3A, in orange and blue, respectively), which also suggested the existence of BOX1-driven oligomers.

These NMR analyses suggested a two-key mechanism whereby Pub1 and Pab1 bind to eIF4G1 causing conformational changes that promote BOX1 self-assembly (Figure 5C). These two RBPs interact with eIF4G1 elements that contact with BOX1 in the free state (Figure 2D). These contacts likely prevent BOX1 aggregation, whereas the coordinated effect of Pub1/Pab1 binding enhances it.

### Pab1-Pub1-eIF4G1 form micrometer-size condensates

**FIGURE 6:**
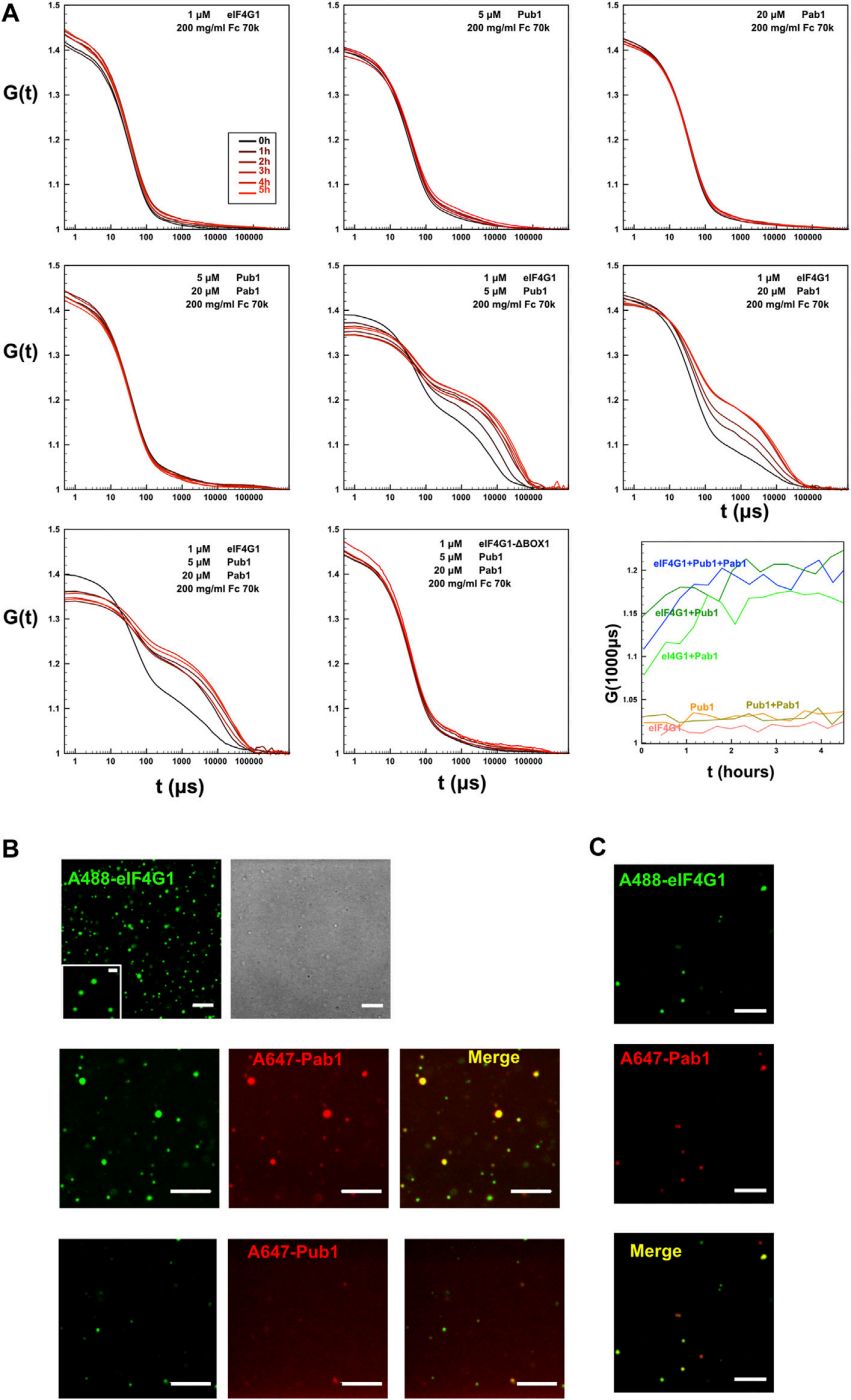
Formation of condensates by Pub1/Pab1/eIF4G1 mixtures in crowding conditions. **(A)** DLS analysis of the indicated individual Pub1 (Pub1 RRM123), Pab1 (Pab1 RRM12), and eIF4G1 (eIF4G1_1-249_ or eIF4G1_1-249_ ΔBOX1) proteins, and of their double and triple mixtures at the concentrations indicated in the figure in the presence of Ficoll (Fc) 70k. Time-dependence (in μs) of the autocorrelation functions G (t) is shown for different protein mixtures and recorded a different time after mixing (panel code shown in the upper left panel). The lower right panel (row 3, column 3) shows the time evolution of the autocorrelation function at 1,000 μs for different mixtures of previous graphs, that correspond to the second phase associated to the aggregates. **(B)** Representative confocal fluorescence microscopic images of ternary mixtures eIF4G1_1-249_ (eIF4G1) labelled with Alexa 488 dye (A488) and Pab1 RRM12 (Pab1) plus Pub1 RRM123 (Pub1). In this ternary mixture, Pab1 plus Pub1 are either unlabeled (row 1, second column) or one of them is labeled with Alexa 647 dye (A647) as indicated in the figure (2^nd^ and 3^rd^ rows), while the other is unlabeled. **(C)** Confocal images of the mixture of eIF4G1_1-249_ (eIF4G1) and Pab1 RRM12 (Pab1), labelled with Alexa 488 and Alexa 647, respectively. In B and C, when present, the final concentration of eIF4G1_1-249_, Pab1 RRM12 and Pub1 RRM123 was 1, 20 and 5 μM, respectively. The concentration of labelled proteins was 1 μM and additional unlabeled protein was added to achieve the indicated final concentration. Scale bars, 5 μm. Inset scale bar, 1 μm. In A, B, and C, samples contained Ficoll 70 (200 g/L) as a crowding agent.

Our NMR analysis suggested that simultaneous binding of Pab1 RRM12, eIF4G1_1-249_ and Pub1 RRM3 has the potential to form high order structures that cannot be detected by this technique because of their large size. To further investigate this possibility, we determined if different Pub1/Pab1/eIF4G1 combinations could form microscopic condensates that might resemble biological ones. For such experiments we used protein concentrations according to the number of molecules per yeast cell (SGD: https://www.yeastgenome.org) and Ficoll 70 (200 g/L) to simulate crowding in the cellular environment. Dynamic light scattering (DLS) indicated that large aggregates were formed in some protein combinations (Figure 6A). In contrast, the individual proteins exhibited autocorrelation functions that did not differ from that of Ficoll-70 alone, suggesting that the individual proteins did not aggregate. Moreover, the curve profiles of the single proteins remained stable for several hours. The Pab1:Pub1 mixture (Figure 6, row 2, column 1) showed the same behavior, but other double protein mixtures and the triple one showed a second phase, evidencing the presence of micrometer-size particles. These particles were present right from the beginning and appeared to reach a maximum within 2 h of mixing (Figure 6A, row 3, column 3). Because, all of these combinations contained eIF4G1 and at least one RNA binding protein, we concluded that the interactions between eIF4G1 and Pab1/Pub1 promoted aggregation; probably by enhancing the intrinsic propensity of BOX1. Consistent with this using the eIF4G1 ΔBOX1 mutant in the triple mixture showed no aggregation (Figure 6, row 3, column 2).

Confocal fluorescence microscopy images of triple mixtures containing Pab1 RRM12, Pub1 RRM123 and Alexa 488 labelled eIF4G1_1-249_ in Ficoll 70 (200 g/L) revealed the presence of discrete rounded particles (∼1 µm and smaller, Figure 6B upper panels). Both, Pab1 and Pub1 were observed to colocalize with eIF4G1 in these assemblies, as observed in fluorescent images in which the proteins were pairwise labelled with spectrally different dyes (Alexa 488 and Alexa 647, Figure 6B middle and lower panels). Similar structures were observed for binary Pab1/eIF4G1 mixtures (Figure 6C), in good agreement with the DLS measurements.

These results showed that the eIF4G1/Pab1/Pub1 mixtures could form crowding-driven structures resembling those previously described for full length Pab1 (Riback et al., 2017) or Pub1 (Kroschwald et al., 2018), but without the requirement for pH or temperature stress.

### eIF4G1 RNA recognition

**FIGURE 7:**
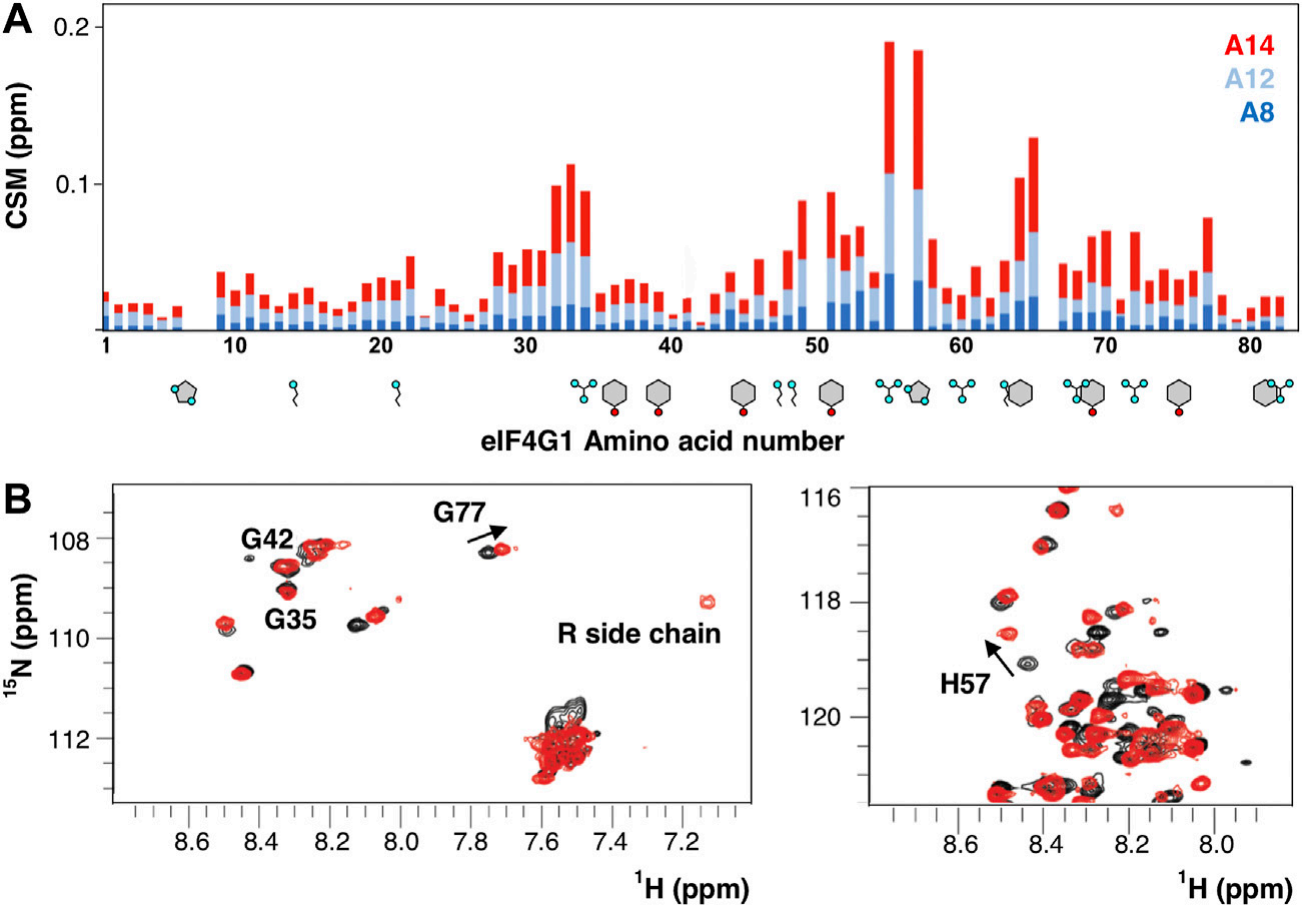
RNA recognition by eIF4G1_1-82_. **(A)** Chemical shift mapping (CSM) of various poly**(A)** probes. Aromatics (Y, H, and F) and basic residues (R and K) are drawn below the sequence. **(B)** Superposition of the ^1^H-^15^N-HSQC of the free (black) and A_14_-bound eIF4G1_1-82_ (red).

Besides protein recognition, eIF4G1 binds RNA using three regions RNA1, RNA2, and RNA3 (Berset et al., 2003; Park et al., 2011). The construct eIF4G1_1-249_ contains one of them (RNA1, eIF4G1_1-82_). We titrate the eIF4G1_1-82_ construct with three poly (A) probes and found that the strength of the interaction is higher with longer oligos (Figure 7A). The chemical shift changes almost doubled when going from A_12_ to A_14_ and a new signal appears in the spectra that corresponds to the side chain Arg guanidinium group (Nε-Hε) (Figure 7B). These spectral changes evidenced that at least one Arg side-chain is directly involved in RNA contacts, slowing down the otherwise rapid solvent exchange in the free protein (no Arg Nε-Hε signals). The interaction with poly(A) maps three regions centered around R_34_, R_55,_ and G_65_, peaking at R_55_PH_57_. In contrast, the canonical RGG box (R_60_GG), a well-known RNA binding motif (Thandapani et al., 2013; Chong et al., 2018; Chowdhury and Jin, 2022), is less affected by binding. In general, the major changes occur in the region with more Arg/Aromatic density and the observed length-dependence of the binding strength probably reflects the simultaneous interactions with various sites (e.g., Arginines) of A_14_.

## Discussion

During the last 40 years stress granules have been described in multiple organisms and upon different stressors, and have been extensively characterized both morphologically and in their composition (Buchan and Parker, 2009; Buchan et al., 2011; Protter and Parker, 2016; Hofmann et al., 2021; Glauninger et al., 2022). However, despite the intensive scientific interest, there is still no clear functional assignment for this type of biomolecular condensates (Glauninger et al., 2022). It is commonly agreed that stress granules appear upon translation arrest, irrespectively the different pathways that lead to it (e.g., eIF2a phosphorylation, mTOR pathway, or targeting eIF4F function). The blockage of the translation machinery leads to polysome disassembly and ribosomes are probably displaced from the mRNA by RBPs such as Pub1, Scd6, Sbp1, etc (all of them characteristic SG markers). Since these RBPs interacts with a wide range of specificity, it is reasonable to think that the RBP load of a given mRNA depend on its length, which correlates well with the higher abundance of long mRNA in SG (Khong et al., 2017; Namkoong et al., 2018; Matheny et al., 2019). However, mRNP assembly does not look on itself sufficient to nucleate biomolecular condensates and evolve them to SG. Individual mRNPs need to bridge together to build a mesh like structure through protein-protein, protein-RNA and RNA-RNA interactions. Knowing the structural features of these interactions is essential to understand the biophysical grounds of SG formation.

Along this line, our work provides an extensive NMR characterization of eIF4G1_1-249_ at the residue level, its complex conformational landscape and its interactions with other SG principal components: the RBPs Pub1 and Pab1. The N-terminus of eIF4G1 contains several short segments (BOXes) that are conserved in the *Saccharomycetales* family and act as short linear interaction motifs (SLiMs) for various purposes: 1) intramolecular and intermolecular self-recognition; 2) specific recognition of RBPs; and 3) RNA recognition.

The eIF4G1_1-249_ conformational ensemble, among the few atomistic models of an IDP (Schwalbe et al., 2014; Cordeiro et al., 2019; Kubáň et al., 2019; Shrestha et al., 2019), shows a high degree of structural variability while maintaining a certain degree of compactness due to long-range contacts among several SLiMs. BOX1 stands out by defining a fuzzy network of π-π and π-cation interactions with RNA1 and BOX3 motifs that mask its prion-like tendency. The sequence of BOX1 shows overlapping Pub1 binding (YNN) and amyloid-like (YYNN) motifs, which are statistically underpopulated in the yeast proteome, but relatively abundant among Pub1 and eIF4G1 binding proteins and in SG core components (Supplementary Figure S6). We speculate that these motifs are SG hallmarks.

Other fundamental questions in the field are what are the mechanisms of nucleation of biomolecular condensates of how these small entities grow to large SG (assuming that both are related) (Glauninger et al., 2022). In the light of our *in vitro* results, we can propose a model pretending to address those questions partially. BOX1 self-assembly propensities are the key feature of the system (Figure 5C). Under non-stress situations, eIF4G1 associates with other translation initiation factors and ribosome 40S subunit to promote translation and BOX1 self-assembly is masked by transient contacts with other regions of the eIF4G1 N-terminal IDP. Translation arrest will depopulate polysomes and Pub1 (and other translational repressors) incorporate into the mRNP. The incoming Pub1 would compete out stabilizing interactions of BOX1, triggering condensation. In this model it is interesting to highlight the role of Pub1 RRM3. The interaction with eIF4G1 would not interfere with RNA recognition. Pub1 is the yeast homolog of TIA-1 and TIAR proteins, which are components of mammalian stress granules (Kedersha et al., 1999). We showed that these this family of proteins has an RRM3 domain with a unique structure (Santiveri et al., 2011). Now our data suggest that this domain (and no other RRMs) can form large assemblies with some eIF4G1 constructs. Finding if these assemblies have some degree of long order would be an important field of study for future research.

## Material and methods

### Cloning, protein expression and purification

Plasmids and proteins used in this work are described in the Supplementary Table S1. DNA fragments corresponding to wild-type constructs of eIF4G1, Pub1, and Pab1 were amplified from *Saccharomyces cerevisiae* genomic DNA using the DNA polymerases KOD or Pfu. These DNA fragments were cloned into a pET28-modified vector that contains an N-terminal thioredoxin A fusion tag, an internal 6xHis tag and a TEV protease site. eIF4G1 mutants were obtained using the Quick-change Lightning Kit and specific DNA primers. Plasmids corresponding to mutant and wild-type proteins were transformed into *E. coli* BL21 (DE3) competent cells and expressed in kanamycin-containing (30 μg/L) LB medium.

For isotope labelling of samples, a K-MOPS derived minimal medium (Neidhardt et al., 1974) was supplemented with ^15^NH_4_Cl (1 g/L) and/or ^13^C-glucose (4 g/L). Cultures of eIF4G1 and its mutants were grown at 37°C until OD_600nm_ = 0.6–0.8, when they were induced with 0.5 µM IPTG for 4 h. Pab1 and Pub1 cultures, after reaching OD_600nm_ = 0.6, were transferred to 25°C for induction with IPTG overnight (12–16 h).

For purification of all recombinant proteins, cell pellets were resuspended in lysis buffer (25 mM potassium phosphate pH 8.0, 300 mM NaCl, 10 mM imidazole and 1 tablet/50 ml of protease inhibitors cocktail), lysed by sonication and cleared by ultracentrifugation. The supernatant was purified by metal affinity chromatography using a HiTrap™ 5 ml column and elution with 25 mM potassium phosphate buffer pH 8.0, containing 300 mM NaCl and 300 mM imidazole. The samples containing the fusion protein were exchanged into 20 mM Tris pH 8.0 (in the case of the Pab1 construct this buffer was supplemented with 1 mM DTT), and digested overnight at 4°C with homemade TEV protease. In the case of the Pub1 and Pab1 constructs, the samples were re-loaded onto the HiTrap nickel column to capture the protease, the cleaved N-terminal part of the fusion protein and the undigested protein. The flow through was further purified by ion exchange using an anion exchanger column (Q 5 ml) for all proteins except for Pub1 RRM3 that was purified with a cation exchange column (SP 5 ml). In either case, proteins were eluted with a linear salt gradient (to 1 M NaCl). In the case of the eIF4G1 construct, we observed that the second nickel column negatively affected protein stability and aggregation; we therefore purified the protein away from the uncleaved protein, thioredoxin A and TEV using a cation exchange column (SP 5 ml). Finally, the purified proteins were concentrated and the buffer was exchanged according to their intended use.

### Small angle X-ray scattering measurements

SAXS experiments were performed using the P12-EMBL beamline at the DESY synchrotron in Hamburg and were analysed with ATSAS software. All data were collected in batch using 25 mM potassium phosphate pH 6.5 and 25 mM NaCl. The concentrations used for analysis of eIF4G1_1-249_ were 15 mg/ml, 10 mg/ml, 8 mg/ml, 5 mg/ml, 3 mg/ml, and 1 mg/ml. The final SAXS curves were generated using PRIMUS and deposited in the SASBDB under the code SASDP88 (https://www.sasbdb.org/data/SASDP88/678c641ui8/).

### NMR: Resonance assignments and relaxation data

All samples were prepared in NMR buffer (25 mM potassium phosphate pH 6.5, 25 or 150 mM NaCl, 1 mM DTT, and 10% D_2_O) and experimental data were acquired at 25°C on a cryoprobe-equipped Bruker AV800 MHz spectrometer. Assignment of the backbone ^1^H, ^15^N and ^13^C atoms was achieved by following the standard methodology. The 3D HNCA, HNCO, HN(CO)CA, CBCA(CO)NH and CBCANH experiments were used for backbone assignment and 3D (H)CCH-TOCSY were recorded to assign side chain resonances [(Sattler et al., 1999) and the references therein]. Protein concentrations ranged between 100–200 μM. The chemical shifts were deposited in the Biomagnetic Resonance Database (BMRB) with codes 28,121 (eIF4G1_1-249_) and 34,517 (eIF4G1_35-49_-Pub1 RRM3). The ^15^N backbone amide relaxation T_1_ and T_2_ parameters were measured with series of ^1^H-^15^N spectra of standard inversion-recovery and Carr-Purcell-Meiboom-Gill sequences (CPMG). NMR spectra were processed using TOPSPIN v4.1 (Bruker) and NMRPipe, and analyses were done with CcpNmr Analysis.

### NMR: Residual dipolar couplings and PRE measurements

The filamentous phage Pf1 was used at a final concentration of 20 mg/ml to induce weak alignment of eIF4G1_1-249_ (200 μM in 25 mM potassium phosphate pH 6.5 and 25 mM NaCl). NMR experiments were carried out at 298 K in a Bruker Avance III 800 MHz spectrometer equipped with a cryogenic triple resonance probe. Two samples (isotropic and anisotropic) were prepared and couplings (J and J + D) were measured with ^15^N-HSQC-DSSE (In Phase Anti Phase IPAP). Experiments were processed using TOPSPIN v2.1 and NMRPipe, and were analyzed with the program CcpNmr Analysis.

For the paramagnetic relaxation enhancement, protein samples from different cysteine-containing eIF4G1_1-249_ mutants (S200C and Q109C) were chemically modified using the following protocol. Mutant protein samples (600–700 µM) were pre-treated with 5 mM DTT for 2 hours at room temperature. The DTT was then eliminated by fast buffer exchange into 25 mM Tris pH 9.0 and 25 mM NaCl using a Nap-5 desalting column. Labelling with 4-(2-Iodoacetamido)-TEMPO was initiated immediately after column elution by adding a tenfold molar excess of the probe dissolved in ethanol (25 mM spin label stock). The reaction was allowed to proceed for 30 min at room temperature in the dark. The excess iodoacetamide label was quenched with 10 mM 2-mercaptoethanol for 10 min, and afterwards the protein adduct was exchanged into 25 mM potassium phosphate pH 6.5, 25 mM NaCl and 1 mM DTT for later use. The NMR samples were prepared in 5 mm tubes sealed in an N_2_ atmosphere to avoid reduction by air, and high resolution ^1^H-^15^N HSQC spectra were recorded for the oxidized state (active spin label). Subsequently, the spin label was reduced with 10 μM ascorbate (Gillespie and Shortle, 1997), and reference ^1^H-^15^N HSQC without paramagnetic relaxation enhancement was recorded. The relaxation effect was calculated as the intensity ratios between peaks in the two spectra.

### Structure calculations of eIF4G1_35-49_-Pub1 RRM3 and the eIF4G BOX3 peptide

The NMR structure of the eIF4G_187-234_ construct was determined from NOE-derived distance restraints (2D NOESY spectrum with 60 ms mixing time) and angular restraints (from 13C chemical shifts and TALOS+) using the program Cyana. Protein assignments were obtained by comparison with other eIF4G1 constructs and confirmed by triple resonance 3D spectra: CBCA(CO)NH, HNCACB and HNCO.

Two different chimeras of Pub1 with eIF4G1_37-51_ were constructed, with eIF4G1_35-49_ fused either to the N- or C- terminus of Pub1 RRM3. Of these constructs, only the N-terminal fusion (eIF4G1_35-49_) proved to have the right topology and the structure was determined using a similar protocol to the analysis of the structure of Pub1 RRM3 (Santiveri et al., 2011) using distance restraints from a 2D NOESY (60 ms mixing time).

### Structure calculation of eIF4G1_1-249_

eIF4G1_1-249_ structures (80,000) were calculated with the program Cyana 3.0 using: 1) experimental NOE-derived distance restraints for the BOX3 region; 2) π- π interactions between Tyr, Phe and Trp; and 3) π-cation interactions between Arg and Tyr/Phe/Trp. The latter two interaction types were referred to as knowledge-based constraints (K-BC) and were included, given the importance of these types of contacts for IDP interactions (see main text for references). For each individual structure calculation, the origin residue (Arg/Tyr/Phe/Trp) was randomly selected (80% probability) and ambiguous restraints were generated for the other interaction partner (Arg/Tyr/Phe/Trp). In this way, each of the individual structure calculations contains a unique set of knowledge-based distance restraints. This protocol ensures high variability by avoiding biases of specific pairwise iterations. Thus, the final interactions present on each conformer are freely selected during the calculations. A similar protocol was followed to calculate the structures (80,000) of eIF4G1_1-249_ dimers, using the same experimental restraints and ambiguous Tyr-Tyr contacts as dimerization driving interactions.

The theoretical PRE-derived intensity ratios were calculated using equations in (Battiste and Wagner, 2000) (Supplementary Figure S5) for the eIF4G1_1-249_ monomers and dimers. Correlation time τ_c_ was estimated from the averaged T_1_/T_2_ and *d* was estimated from the distances between amide backbones (N) and Q109/S200 side-chains (Cβ). We next used a home-made greedy algorithm to select the ensembles that better reproduced the PRE profiles. The algorithm calculates the residual to the experimental data (Q109 and S200) and chooses, as a seed, the conformer that better agrees (lower sum of residuals) with the data. In the next steps, the residuals were computed across 2, 3,.., n structures always choosing the combination with minimal violations. The procedure was repeated until the ensemble size reached 2,000 conformers (∼2.5% of the original). The first 500 structures were included in the final eIF4G1_1-249_ monomer and eIF4G1_1-249_ dimer ensembles **(**PRE-derived).

We used the EOM protocol to fit the SAXS data. Pools of theoretical SAXS curves were constructed for the eIF4G1_1-249_ monomers (2,000 conformers) and dimers (2,000 conformers). These two pools were combined with the genetic algorithm in the EOM program to model the curve with a fixed size ensemble (50 structures). The percentages of each pool were freely selected by the algorithm and the procedure. The procedure was repeated 10 times, obtaining a 500-member set. It should be noted that some of the structures are repeated between individual EOM calculations. The theoretical values of the PREs, and other structural properties, were calculated as averages across the different ensembles using home-made perl scripts.

### Dynamic light scattering

The DLS measurements were carried out at 25°C in a DynaPro Titan (Wyatt Technologies) instrument and were analysed with Dynamics V6 software. Protein mixtures (eIF4G1_1-249_, eIF4G1_1-249_ ∆BOX1, Pub1 RRM123 and Pab1 RRM12) were prepared from extensively centrifuged stocks (>1 h at 15,000 RPM; 4°C), and were filtered (0.22 μm) in PBS buffer and 200 g/L Ficoll 70 stock. Samples were mixed thoroughly and placed in a plastic cuvette (Eppendorf) for measurements. Individual correlation curves were recorded (10 acquisitions of 10s) every 15 min over a 5 h period.

### Confocal microscopy

eIF4G1_1-249_, Pab1 RRM12, and Pub1 RRM123 proteins were purified as described above and were labelled with Alexa Fluor 488 or Alexa Fluor 647 carboxylic acid succinimidyl ester dyes (Molecular Probes), using protein to probe ratios of 1:3. The coupling reaction was carried out in the dark in PBS (pH 7.4) buffer for 30 min on ice and the unreacted probe was removed by size exclusion chromatography using a Nap-5 column. Samples for visualization were prepared by mixing eIF4G1_1-249_ with Pub1 RRM123 and Pab1 RRM12 in different combinations and at concentrations of 1, 5 and 20 μM respectively, in PBS, 0.1 mM DTT (pH 7.4) buffer and 200 g/L Ficoll 70. Samples, that contain 1 μM of fluorescently labelled protein (Alexa Fluor 488-labelled eIF4G1 and/or Alexa Fluor 647-labelled Pub1 or Pab1) for visualization, were placed in silicone chambers that were glued to coverslips and were visualized with Leica TCS SP2 or TCS-SP5 inverted confocal microscopes with a HCX PL APO ×63 oil immersion objective (N.A. = 1.4; Leica, Mannheim, Germany). Alexa 488 and Alexa 467 were excited using 488 and 633 nm laser excitation lines, respectively. The concentration of the various Alexa-labelled proteins was kept at 1 μM and the solution was supplemented with unlabeled proteins to reach the above-mentioned concentrations. Various images were registered for each sample, corresponding to different observation fields.

## Data Availability

The datasets presented in this study can be found in online repositories. The names of the repository/repositories and accession number(s) can be found below: http://www.wwpdb.org/, 6Z29. SAXS data was deposited in the SASBDB under the code SASDP88. Chemical shifts were deposited in the Biomagnetic Resonance Database (BMRB) with codes 28121 and 34517. The structural ensembles were deposited in the Protein Ensemble Database (PED) with the codes PED00225 and PED00226.
