# Supplementary material for: eIF4G1 N-terminal intrinsically disordered domain is a multi-docking station for RNA, Pab1, Pub1, and self-assembly
Source: Front Mol Biosci. 2022 Sep 23;9:986121. doi: 10.3389/fmolb.2022.986121 (PMC9537944; doi:10.3389/fmolb.2022.986121)
Supplement: Supplementary file 1 [file DataSheet1.docx]

**Supplementary data**

**eIF4G1 N-terminal intrinsically disordered domain is a multi-docking station for RNA, Pab1, Pub1 and self-assembly**

Belén Chaves-Arquero, Santiago Martínez-Lumbreras, Nathalie Sibille, Pau Bernadó, Mª Ángeles Jiménez and José Manuel Pérez-Cañadillas^*^.

**Supplementary Figure 1.** Stability of eIF4G1_1-187_ and eIF4G1_1-249_ constructs at equivalent concentrations (50 μM) and under different buffer conditions. Samples were incubated at 25 °C for 0, 1, 4, 5, or 8 days in 25 mM sodium acetate (pH 4.5, 5.5) or potassium phosphate buffer (pH 6.5) containing 150 mM NaCl and 0.0005% NaN_3_. Samples were then electrophoresed and the gels were labelled with Coomassie Blue. The construct lacking the BOX3 element (eIF4G1_1-187_) is less stable than that with this element (eIF4G1_1-187_).

**Supplementary Figure 2. (A)** Schematic view of the chemical process of deamidation of asparagines. The pentacyclic intermediate could evolve to isoD or D, but only the first pathway was detected for eIF4G1_1-249_. **(B)** ^15^N planes of various triple resonance experiments show crosspeaks within the isoD_41_-G_42_-S_43_ segment indicating the sequential connectivities. The expected magnetization transfers for each spectrum type are shown above with arrows colored in the same color as the experimental data. The sequential connections are interrupted in HNCA, HNCO and HNCACO due to the β-configuration of the backbone at isoD_41_.


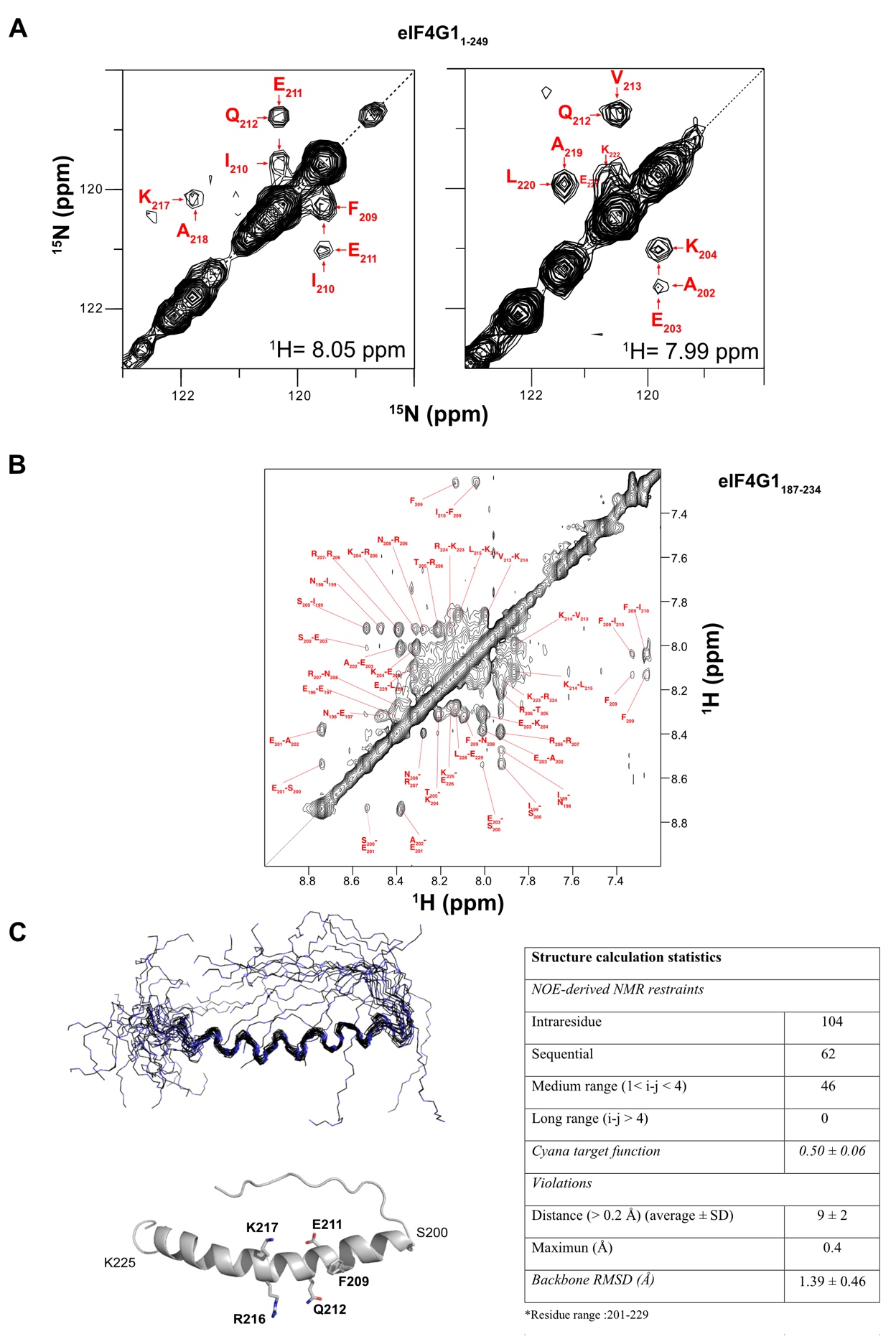


**Supplementary Figure 3. (A)** The ^15^N-^15^N planes in the 3D ^1^H-^15^N-HSQC-NOESY-^1^H ^15^N-HSQC spectra of eIF4G1_1-249_ show the characteristic sequential HN-HN NOEs between consecutive residues of the α-helix in BOX3. **(B)** Detail of the HN-HN NOEs in the 2D NOESY of eIF4G1_187-234_. **(C)** The NMR structure of this eIF4G1_187-234_ construct forms a continuous, slightly curved, α-helix with 7-turns in which the conserved residues (F209, E211, Q212, R216 and K217) are located in the middle. Structure calculation statistics are shown on the right.


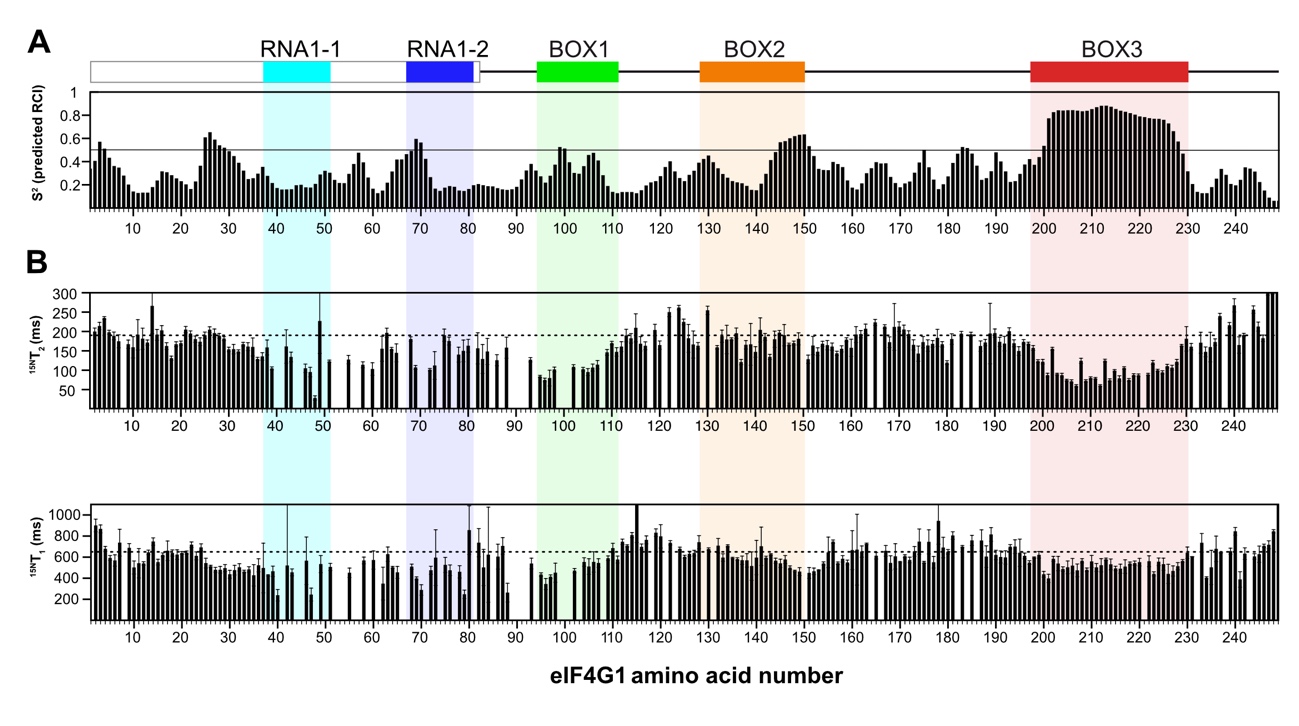


**Supplementary Figure 4**. **(A)** Random coil index (RCI) S2 values predicted from chemical shifts of eIF4G1_1-249_ using the program Camshift. **(B)** Experimental values of ^15^N T_1_ and T_2_ at 25 ºC in an Bruker AV800 spectrometer. Amino acids located in eIF4G1_1-249_ conserved regions are indicated in color.

**Supplementary Figure 5.** The theoretical intensity ratios of oxidized (ox) to reduced (red) forms of the spin-labeled eIF4G1_1-249_ mutants were computed as previously described in (Battiste and Wagner, 2000) using eq. 1 and 2. Proton R_2_ relaxation was estimated from the average of cross peak line widths at half-height (eq. 3) and correlation time (τ_c_) from the average T^N^_1_/T^N^_2_ values (Figure 2A middle panel). The functional dependence of eq.1 on distance is shown in the graph on the right. The vertical line at 30 Å marks the Paramagnetic Relaxation Enhancement (PRE) upper limit used for all the experimental I_ox_/I_red_ values below 0.8.


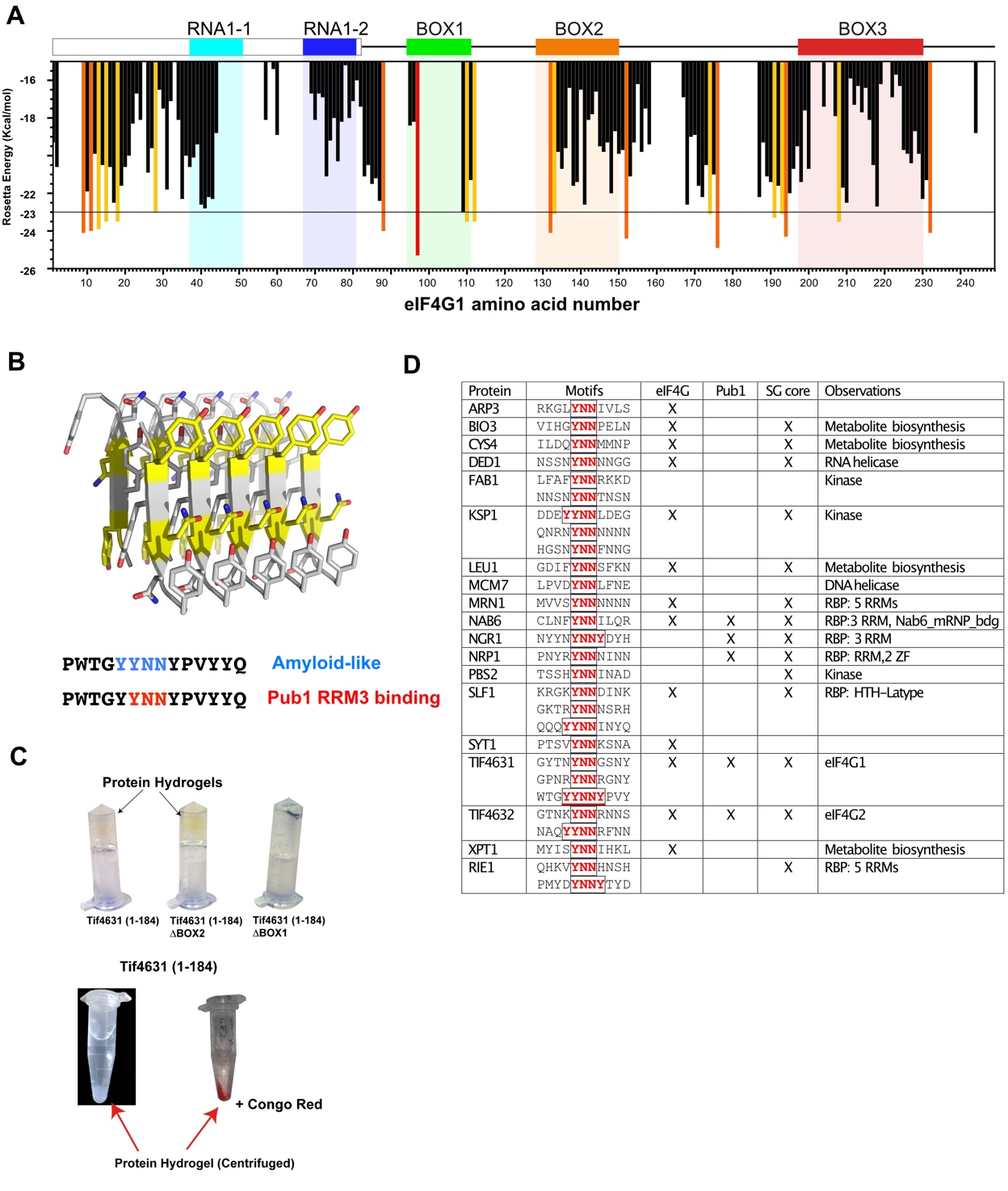


**Supplementary Figure 6. (A)** Fibrillation propensity of hexapeptide sequences in eIF4G1_1-249_ calculated using [ZIPPERDB](https://services.mbi.ucla.edu/zipperdb/). Segments whose Rosetta models have energies below -23 Kcal/mol are coloured in yellow (-23.0 to -24. Kcal/mol), orange (-24.0 to -25.0 Kcal/mol) and red (< -25.0 Kcal/mol). See program reference for specific details. **(B)**. Amyloid model for eIF4G1_96-102_. **(C)** Hydrogel forming capacity of aged samples of different eIF4G1 constructs. Freshly purified samples ( ~200 μM) in 25 mM potassium phosphate buffer (pH 6.5); 25 mM NaCl were simultaneously incubated at 4ºC for 2 weeks and visually checked for jellification. Congo-red dye (at ~0.1 mg/ml) was added to jellified samples, incubate for 30 min at room temperature and centrifuge at 10000 RPMs to separate the gel fraction that is enriched in the dye. **(D)** Distribution of the YYNN and YNN motifs in the S. cerevisiae proteome. Columns show the sequence alignments, presence on eIF4G, in Pub1 interactomes (from the SGD database), and in the stress granule (SG) core particles (Jain et al., 2016).

**Supplementary Figure 7.** The left panel shows the evolution of Residual PRE violations (as defined in materials and methods) as a function of the eIF4G1_1-249_ ensemble size. The right panel shows the comparison of the calculated PRE values from the PRE-selected (red) and the EOM selected ensembles (orange). See materials and methods for further details.

**Supplementary Figure 8. (A)** Sequence of the eIF4G1_35-49_-Pub1 RRM3 chimera. **(B)** Backbone superposition of the 20 conformers of the NMR structure of the eIF4G1-Pub1 RRM3 chimera (red corresponds to eIF4G1) and the table with structure calculation statistics. **(C**) Comparison of the yeast Pub1 RRM3-eIF4G1 and the human PABP RRM2-eIF4G1 (Safaee et al., 2012) binding modes.


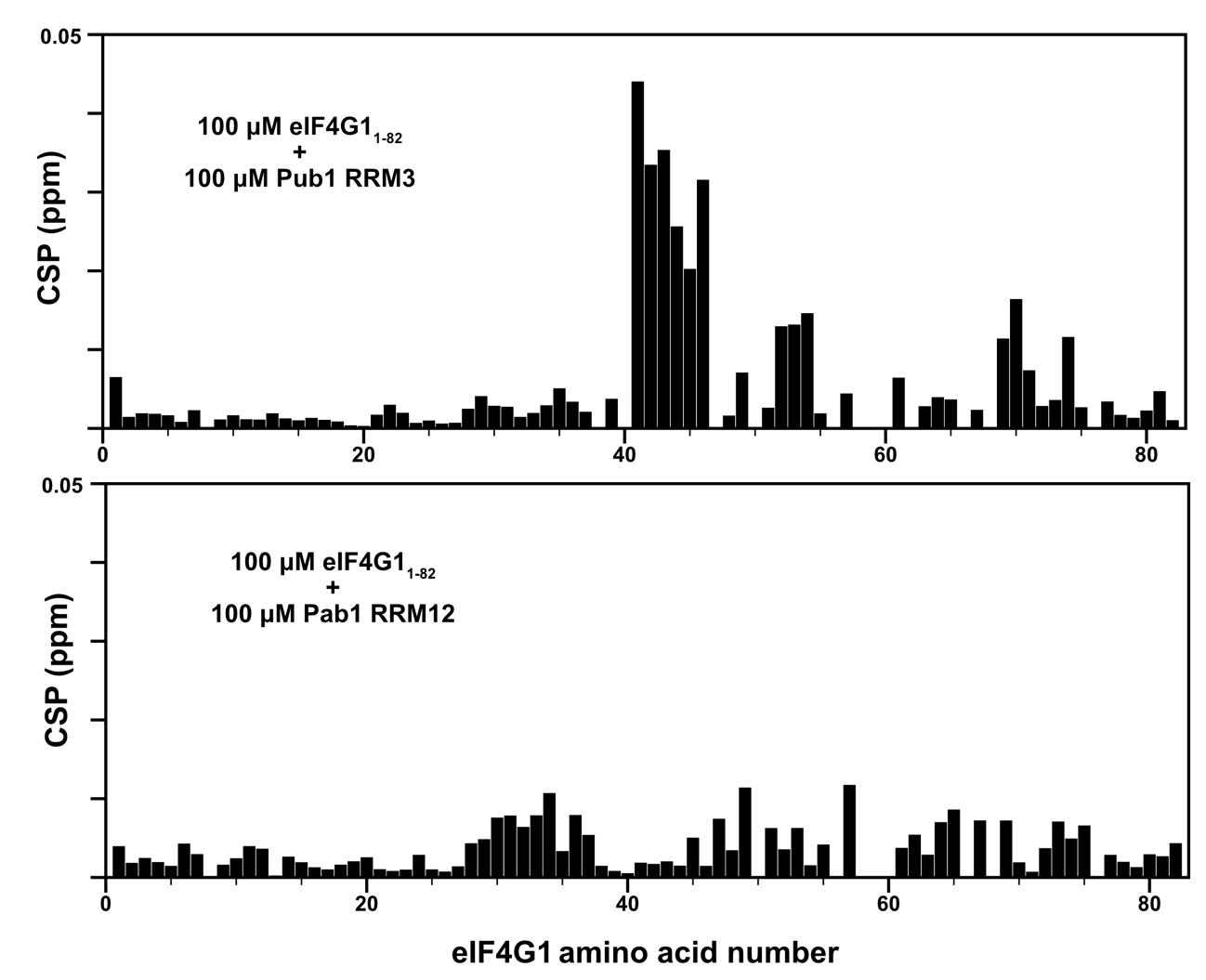


**Supplementary Figure 9.** Sequence dependence histogram of chemical shift perturbations of the ^1^H-^15^N HSQC signals of the eIF4G1_1-82_ upon titration of equivalent amounts of unlabeled Pub1 RRM3 (upper histogram) and Pab1 RRM12 (lower histogram). NMR experiments were performed under equivalent condictions of buffer, temperature and protein concentrations.

**
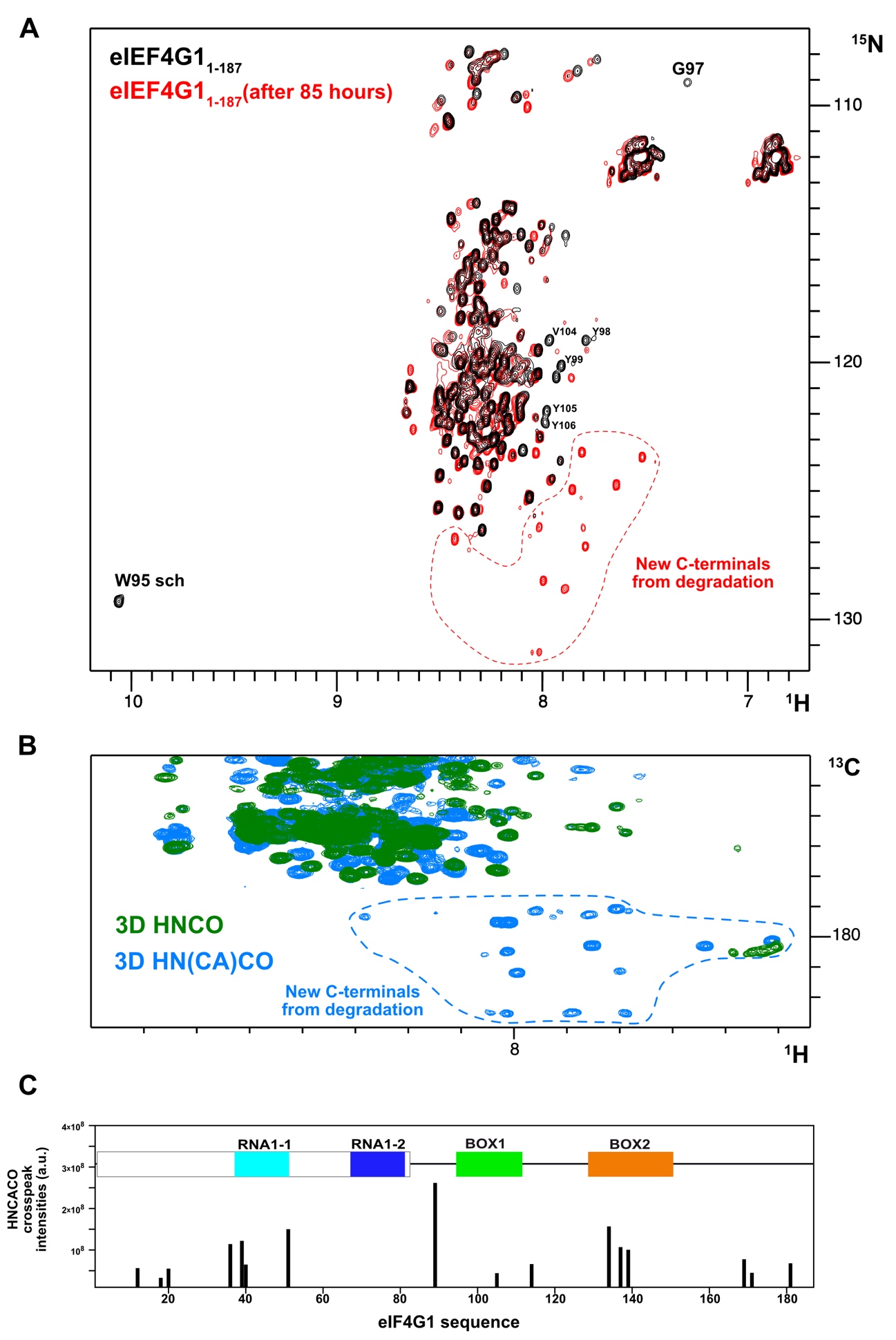
**

**Supplementary Figure 10.** NMR characterization of the eIF4G1_1-184_ degradation. **A**. Comparison between ^1^H-^15^N HSQC from fresh (black) and aged (red) samples. The two experiments were registered after 85 hours at 25ºC. The signals corresponding to BOX1 disappear in the aged sample, some of them are labelled. New C-terminal are enclosed in the spectrum of the aged sample. **B**. Projection along the ^15^N axis of the 3D HNCO and 3D HNCACO to show the crosspeaks corresponding to the new C-terminal arising from degradation. **C**. Sequence histogram of the new C-terminal 3D HNCACO crosspeaks with their intensities. The strongest signal correspons to the new Asn 89 C-terminus (that effectively disconnect BOX1 from RNA1_1 and RNA1_2 sequences). The samples shows jellification after the experiments similar to those in supplementary figure 6C.

**Supplementary table 1**

| **REAGENT or RESOURCE** | | | | **SOURCE** | | | | | | | **IDENTIFIER/SEQUENCE** | |  |  |
| --- | --- | --- | --- | --- | --- | --- | --- | --- | --- | --- | --- | --- | --- | --- |
| Bacterial and Virus Strains | | | | | | | | | | | | |  |  |
| *E. Coli*: BL21(DE3) chemically competent cells | | | | Stratagene | | | | | | | 200131 | |  |  |
| *E. Coli: XL10-Gold* chemically competent cells | | | | Stratagene | | | | | | | 200314 | |  |  |
| Chemicals | | | | | | | | | | | | |  |  |
| Filamentous phage Pf1 | | | | ASLA biotech | | | | | | | P-50-RNA | |  |  |
| Ficoll PM70 | | | | GE Healthcare | | | | | | | Product:17031005 | |  |  |
| 4-(2-Iodoacetamido)-TEMPO | | | | Sigma-Aldrich | | | | | | | CAS:25713-24-0 | |  |  |
| Deuterium Oxide D_2_O | | | | Euriso-top | | | | | | | CAS:7789-20-0 | |  |  |
| Lysozyme from chicken egg white | | | | Sigma-Aldrich | | | | | | | CAS:12650-88-3 | |  |  |
| Protease Inhibitors cOmplete ultra tablets | | | | Roche | | | | | | | REF. 05892791001 | |  |  |
| Isopropylthio-ß-galactoside, IPTG | | | | Generon | | | | | | | Cat# GF101-01 | |  |  |
| 1,4-Dithiothreitol, DTT | | | | Sigma-Aldrich | | | | | | | CAS:3483-12-3 | |  |  |
| 2-Mercaptoethanol | | | | Sigma-Aldrich | | | | | | | CAS:60-24-2 | |  |  |
| Imidazole, 99% | | | | ACROS Organics^TM^ | | | | | | | CAS:288-32-4 | |  |  |
| Sodium chloride | | | | Fisher Chemical | | | | | | | CAS:7647-14-5 | |  |  |
| Potassium Phosphate Dibasic | | | | Fisher BioReagents | | | | | | | CAS:7758-11-4 | |  |  |
| Potassium Phosphate Monobasic | | | | ACROS Organics^TM^ | | | | | | | CAS:7778-77-0 | |  |  |
| Yeast Extract | | | | CONDA pronadisa | | | | | | | Cat#1702-00 | |  |  |
| Tryptone | | | | CONDA pronadisa | | | | | | | Cat#1612-00 | |  |  |
| Ammonium chloride (15N, 99%) | | | | Cambridge Isotope Lab. | | | | | | | CAS:39466-62-1 | |  |  |
| D-Glucose (U-13C6, 99%) | | | | Cambridge Isotope Lab. | | | | | | | CAS:110187-42-3 | |  |  |
| MOPS | | | | Sigma-Aldrich | | | | | | | CAS:1132-61-2 | |  |  |
| Tricine | | | | Sigma-Aldrich | | | | | | | CAS:5704-04-1 | |  |  |
| Magnesium sulfate | | | | Fluka | | | | | | | CAS:17830-18-1 | |  |  |
| Calcium chloride | | | | Panreac | | | | | | | Cod:141219 | |  |  |
| Ferrous chloride | | | | Probys | | | | | | | Cod:53198 | |  |  |
| Cobalt chloride | | | | Fluka | | | | | | | CAS:7791-13-1 | |  |  |
| Cooper chloride | | | | UCB | | | | | | | Cod:1277 | |  |  |
| Manganese chloride | | | | UCB | | | | | | | Cod:1436 | |  |  |
| Zinc chloride | | | | Fluka | | | | | | | CAS:7646-85-7 | |  |  |
| Sodium molybdate dehydrate | | | | Sigma-Aldrich | | | | | | | CAS:10102-40-6 | |  |  |
| Thiamine hydrochloride | | | | Sigma-Aldrich | | | | | | | CAS:67-03-8 | |  |  |
| d-Biotine | | | | Sigma-Aldrich | | | | | | | CAS:58-85-5 | |  |  |
| Choline chloride | | | | Sigma-Aldrich | | | | | | | CAS:67-48-1 | |  |  |
| Folic Acid | | | | Sigma-Aldrich | | | | | | | CAS:59-30-3 | |  |  |
| Niacinamide | | | | Sigma-Aldrich | | | | | | | CAS:98-92-0 | |  |  |
| d-pantothenic | | | | Sigma-Aldrich | | | | | | | CAS:137-08-6 | |  |  |
| Pyridoxal | | | | Sigma-Aldrich | | | | | | | CAS:65-22-5 | |  |  |
| Riboflavin | | | | Sigma-Aldrich | | | | | | | CAS:83-88-5 | |  |  |
| Tris | | | | Fisher | | | | | | | CAS:7786-1 | |  |  |
| Kanamycin sulfate | | | | Calbiochem | | | | | | | Cat#420411 | |  |  |
| Boric acid | | | | Sigma-Aldrich | | | | | | | CAS:10043-35-3 | |  |  |
| PIPES | | | | Sigma-Aldrich | | | | | | | CAS:5625-37-6 | |  |  |
| Potassium chloride | | | | Fisher | | | | | | | CAS:7447-40-7 | |  |  |
| Dimethyl sulfoxide, DMSO | | | | Carlo Erba | | | | | | | Cod:445103 | |  |  |
| Tris hydroxymethyl aminomethane hydrochloride | | | | ACROS Organics^TM^ | | | | | | | CAS:1185-53-1 | |  |  |
| Sodium dodecyl sulfate, SDS | | | | Silga-Aldrich | | | | | | | CAS:151-21-3 | |  |  |
| Acrylamide | | | | Scharlau | | | | | | | CAS:79-06-1 | |  |  |
| N,N,N’,N’-tetra methyl-ethylenediamine, TEMED | | | | Sigma-Aldrich | | | | | | | CAS:110-18-9 | |  |  |
| Agarose LM | | | | Pronadisa | | | | | | | Cat#8051 | |  |  |
| Alexa Fluor 488 | | | | Invitrogen | | | | | | | Cat#A-11001 | |  |  |
| Alexa Fluor 647 | | | | Invitrogen | | | | | | | Cat#A-21235 | |  |  |
| Agarose EEO | | | | Pronadisa | | | | | | | Cat#8022 | |  |  |
| TEV protease | | | | Homemade | | | | | | | N/A | |  |  |
| Critical Commercial Assays | | | | | | | | | | | | |  |  |
| Taq Master Kit | Jean Bioscience | | | | | Cat#PCR-101L | | | | | |  |  |  |
| Kit PCR Purification | Jean Bioscience | | | | | Cat#PP-2015 | | | | | |  |  |  |
| New Builder Hifi DNA Assembly Master Mix | BioLabs | | | | | Cat#D2621L | | | | | |  |  |  |
| Bug Buster Master Mix | Millipore | | | | | Cat#71456 | | | | | |  |  |  |
| QuikChange Lightning Kit | Agilent genomics | | | | | Ref.210515 | | | | | |  |  |  |
| DNA polymerase KOD | Novagen | | | | | Ref. 71085-3 | | | | | |  |  |  |
| DNA polymerase Pfu | Promega | | | | | Ref. M774A | | | | | |  |  |  |
| Fast n-Easy Plasmid Miniprep Kit | Jean Bioscience | | | | | Cat#PP-204S | | | | | |  |  |  |
| Deposited Data | | | | | | | | | | | | |  |  |
| PDBs | eIF4G1_35-49_-Pub1 RRM3 | | | | | | 6Z29 | | | | | |  |  |
| BMRB | eIF4G1 1-249 | | | | | | 28121 | | | | | |  |  |
| BMRB | eIF4G1_35-49_-Pub1 RRM3 | | | | | | 34517 | | | | | |  |  |
| Oligonucleotides | | | | | | | | | | | | |  |  |
| PUB1_315_FW | SIGMA | | | | CGCGGATCCCAGACCATTGGTTTACCTCCTCAAGTAAATCCTCAAGC | | | | | | | |  |  |
| PUB1_414_RV | SIGMA | | | | GCGCTCGAGTTATCTTTCCTTACCCCAACCGGTTCTCAACTTTCTGCG | | | | | | | |  |  |
| PAB1_1_FW | IDT | | | | GCGCGGATCCATGGCTGATATTACTGATAAGACAGCTGAACAATTGG | | | | | | | |  |  |
| PAB1_213_FW | MACROGEN | | | | ACTCTCAATTGGAATAAACTAAGGCACATTACAC | | | | | | | |  |  |
| PAB1_213_RV | MACROGEN | | | | GTAATGTGCCTTAGTTTATTCCAATTGAGAGTC | | | | | | | |  |  |
| TIF4631_1_FW | IDT | | | | CGCGGATCCATGACAGCAGAAACTGCTCACCCGACACAATCTGC | | | | | | | |  |  |
| TIF4631_403_ST_RV | IDT | | | | CGCGCTCGAGTTAATCAGTTGTAGTTTCGATTTCAGCTTCAAGTCC | | | | | | | |  |  |
| TIF4631_82_stop_RV | IDT | | | | CGCGCTCAGATTATCTGAAACTACCGCCACC | | | | | | | |  |  |
| TIF4631_83_FW | IDT | | | | CGCGGATCCGGTGGACACATGGGAGCCAACAGC | | | | | | | |  |  |
| TIF4631_187_FW | IDT | | | | CGCGGATCCACTAATGACTCTAAGGCCAGTTCTG | | | | | | | |  |  |
| TIF4631_82_STBAM_FW | IDT | | | | GGCGGTAGTTTCAGATAAGGATCCATGGGAGCCAACAGC | | | | | | | |  |  |
| TIF4631_82_STBAM_RV | IDT | | | | GCTGTTGGCTCCCATGGATCCTTATCTGAAACTACCGCC | | | | | | | |  |  |
| TIF4631_188STBAM_FW | IDT | | | | CTTCTACTCCAACTCCTTAAGGATCCACTAATGACTCTAAGG | | | | | | | |  |  |
| TIF4631_188STBAM_RV | IDT | | | | CCTTAGAGTCATTAGTGGATCCTTAAGGAGTTGGAGTAGAAG | | | | | | | |  |  |
| TIF4631_W95A_FW | IDT | | | | GCTCAAACGTGCCAGCGACTGGTTACTATAATAAC | | | | | | | |  |  |
| TIF4631_W95A_RV | IDT | | | | GTTATTATAGTAACCAGTCGCTGGCACGTTTGAGC | | | | | | | |  |  |
| TIF4631_F98_99A_FW | IDT | | | | GTGCCATGGACTGGTGCCGCTAATAACTACCCCG | | | | | | | |  |  |
| TIF4631_F98_99A_RV | IDT | | | | CGGGGTAGTTATTAGCGGCACCAGTCCATGGCAC | | | | | | | |  |  |
| TIF4631_F105_106A_2_FW | IDT | | | | CTATAATAACTACCCCGTTGCCGCCCAGCCCCAGCAA | | | | | | | |  |  |
| TIF4631_F105_106A_2_RV | IDT | | | | TTGCTGGGGCTGGGCGGCAACGGGGTAGTTATTATAG | | | | | | | |  |  |
| TIF4631_DBOX1_FW | IDT | | | | CCAACAGCTCAAACGTGGCGG | | | | | | | |  |  |
| TIF4631_DBOX1_RV | IDT | | | | CCGCCACGTTTGAGCTGTTGG | | | | | | | |  |  |
| TIF4631_DBOX2_FW | IDT | | | | CCAATTCCTGTCGAAGAGAAGAAAGCCAAGCTACAGTCTCAGG | | | | | | | |  |  |
| TIF4631_DBOX2_RV | IDT | | | | CCTGAGACTGTAGCTTGGCTTTCTTCTCTTCGACAGGAATTGG | | | | | | | |  |  |
| TIF4631_305ST_RV | IDT | | | | GCGCTCGAGTTACTTCAAACGTTCAGCAAAGGTTAAC | | | | | | | |  |  |
| TIF4631_348ST_RV | IDT | | | | GCGCTCGAGTTATTTAACCTGTTCACTGGGAGGC | | | | | | | |  |  |
| TIF4631_250ST_FW | MACROGEN | | | | GTGGAAGAGAAGTAATCGGACAAACCTG | | | | | | | |  |  |
| TIF4631_250ST_RV | MACROGEN | | | | GGTTTGTCCGATTACTTCTCTTCCACATTTTCTGG | | | | | | | |  |  |
| TIF4631_274ST_FW | MACROGEN | | | | GAGCCAGAAGTTAAGTAAGAAACTCCAGCTGAAG | | | | | | | |  |  |
| TIF4631_274ST_RV | MACROGEN | | | | CAGCTGGAGTTTCTTACTTAACTTCTGGCTCAGC | | | | | | | |  |  |
| TIF4631_Q109C_FW | MACROGEN | | | | TACCAGCCCTGCCAAATGGCGGCC | | | | | | | |  |  |
| TIF4631_Q109C_RV | MACROGEN | | | | CCGCCATTTGGCAGGGCTGGTAGTAAACG | | | | | | | |  |  |
| TIF4631_S200C_FW | MACROGEN | | | | CTGAAGAAAATATATGTGAAGCTGAAAAGACAAG | | | | | | | |  |  |
| TIF4631_S200C_RV | MACROGEN | | | | CTTTTCAGCTTCACATATATTTTCTTCAGAACTGG | | | | | | | |  |  |
| TIF4631_235ST_FW | MACROGEN | | | | GGTTCTAGTGGCAACTAAAATATTCCAATGAAGACTACC | | | | | | | |  |  |
| TIF4631_235ST_RV | MACROGEN | | | | CTTCATTGGAATATTTTAGTTGCCACTAGAACCTTCAAG | | | | | | | |  |  |
| TIF4631_199_FW | MACROGEN | | | | ATTTCCAGGGATCCATATCTGAAGCTGAAAAGACAAGAAG | | | | | | | |  |  |
| Recombinant DNA plasmids | | | | | | | | | | | | |  |  |
| pET28_txaHTEV_PUB1_R3 | | | (Santiveri et al., 2011) | | | | | | | N/A | | | | |
| pET28_txaHTEV_PAB1_R12 | | | This paper | | | | | | | N/A | | | | |
| pET28_txaHTEV_eIF4G1_1-402_ | | | (Santiveri et al., 2011) | | | | | | | N/A | | | | |
| pET28_txaHTEV_eIF4G1_1-348_ | | | This paper | | | | | | | N/A | | | | |
| pET28_txaHTEV_eIF4G1_1-305_ | | | This paper | | | | | | | N/A | | | | |
| pET28_txaHTEV_eIF4G1_1-249_ | | | This paper | | | | | | | N/A | | | | |
| pET28_txaHTEV_eIF4G1_1-249_ Q109C | | | This paper | | | | | | | N/A | | | | |
| pET28_txaHTEV_eIF4G1_1-249_ S200C | | | This paper | | | | | | | N/A | | | | |
| pET28_txaHTEV_eIF4G1_1-249_ (∆Box1) | | | This paper | | | | | | | N/A | | | | |
| pET28_txaHTEV_eIF4G1_1-184_ | | | (Santiveri et al., 2011) | | | | | | | N/A | | | | |
| pET28_txaHTEV_eIF4G1_1-184_ W95A | | | This paper | | | | | | | N/A | | | | |
| pET28_txaHTEV_eIF4G1_1-184_Y98A/Y99A | | | This paper | | | | | | | N/A | | | | |
| pET28_txaHTEV_eIF4G1_1-184_Y105A/Y106A | | | This paper | | | | | | N/A | | | | |  |
| pET28_txaHTEV_eIF4G1_1-184_ (∆Box1) | | | This paper | | | | | | | N/A | | | | |
| pET28_txaHTEV_eIF4G1_1-184_ (∆Box2) | | | This paper | | | | | | | N/A | | | | |
| pET28_txaHTEV_eIF4G1_1-82_ | | | (Santiveri et al., 2011) | | | | | | | N/A | | | | |
| pET28_txaHTEV_eIF4G1_187-234_ | | | This paper | | | | | | | N/A | | | | |
| pET28_txaHTEV_eIF4G1_35-49_-Pub1 RRM3 | | | This paper | | | | | | | N/A | | | | |
| pET28_txaHTEV_ Pub1 RRM3-eIF4G1_35-49_ | | | This paper | | | | | | | N/A | | | | |
| *Peptides and Recombinant proteins* | | | | | | | | | | | | |  |  |
| Peptide eIF4G1 BOX1 | Caslo | | | | | | NVPWTGYYNNYPVYYQPQQMAAKKK | | | | | |  |  |
| Peptide eIF4G1 RNA1_1 | Caslo | | | | | | GYTNYNNGSYNTQKK | | | | | |  |  |
| Peptide eIF4G1 RNA1_2 | Caslo | | | | | | GPNRYNNRGNYNGGG | | | | | |  |  |
| Peptide eIF4G1 BOX2 | Caslo | | | | | | SPVPTKIEITTKSGEHLDLKEQHKAKLQSQERS | | | | | |  |  |
| Recombinant eIF4G1_1-402_ | (Santiveri et al., 2011) | | | | | | gsMTDETAHPTQSASKQESAALKQTGDDQQESQQQRGYTNYNNGSNYTQKKPYNSNRPHQQRGGKFGPNRYNNRGNYNGGGSFRGGHMGANSSNVPWTGYYNNYPVYYQPQQMAAAGSAPANPIPVEEKSPVPTKIEITTKSGEHLDLKEQHKAKLQSQERSTVSPQPESKLKETSDSTSTSTPTPTPSTNDSKASSEENISEAEKTRRNFIEQVKLRKAALEKKRKEQLEGSSGNNNIPMKTTPENVEEKGSDKPEVTEKTKPAEEKSAEPEVKQETPAEEGEQGEKGQIKEESTPKVLTFAERLKLKKQQKEREEKTEGKENKEVPVQEETKSAIESAPVPPSEQVKEETEVAETEQSNIDESATTPAIPTKSDEAEAEVEAEAGDAGTKIGLEAEIETTTD | | | | | |  |  |
| Recombinant eIF4G1_1-348_ | This paper | | | | | | gsMTDETAHPTQSASKQESAALKQTGDDQQESQQQRGYTNYNNGSNYTQKKPYNSNRPHQQRGGKFGPNRYNNRGNYNGGGSFRGGHMGANSSNVPWTGYYNNYPVYYQPQQMAAAGSAPANPIPVEEKSPVPTKIEITTKSGEHLDLKEQHKAKLQSQERSTVSPQPESKLKETSDSTSTSTPTPTPSTNDSKASSEENISEAEKTRRNFIEQVKLRKAALEKKRKEQLEGSSGNNNIPMKTTPENVEEKGSDKPEVTEKTKPAEEKSAEPEVKQETPAEEGEQGEKGQIKEESTPKVLTFAERLKLKKQQKEREEKTEGKENKEVPVQEETKSAIESAPVPPSEQVK | | | | | |  |  |
| Recombinant eIF4G1_1-305_ | This paper | | | | | | gsMTDETAHPTQSASKQESAALKQTGDDQQESQQQRGYTNYNNGSNYTQKKPYNSNRPHQQRGGKFGPNRYNNRGNYNGGGSFRGGHMGANSSNVPWTGYYNNYPVYYQPQQMAAAGSAPANPIPVEEKSPVPTKIEITTKSGEHLDLKEQHKAKLQSQERSTVSPQPESKLKETSDSTSTSTPTPTPSTNDSKASSEENISEAEKTRRNFIEQVKLRKAALEKKRKEQLEGSSGNNNIPMKTTPENVEEKGSDKPEVTEKTKPAEEKSAEPEVKQETPAEEGEQGEKGQIKEESTPKVLTFAERLK | | | | | |  |  |
| Recombinant eIF4G1_1-249_ | This paper | | | | | | gsMTDETAHPTQSASKQESAALKQTGDDQQESQQQRGYTNYNNGSNYTQKKPYNSNRPHQQRGGKFGPNRYNNRGNYNGGGSFRGGHMGANSSNVPWTGYYNNYPVYYQPQQMAAAGSAPANPIPVEEKSPVPTKIEITTKSGEHLDLKEQHKAKLQSQERSTVSPQPESKLKETSDSTSTSTPTPTPSTNDSKASSEENISEAEKTRRNFIEQVKLRKAALEKKRKEQLEGSSGNNNIPMKTTPENVEEK | | | | | |  |  |
| Recombinant eIF4G1_1-249_ Q109C | This paper | | | | | | gsMTDETAHPTQSASKQESAALKQTGDDQQESQQQRGYTNYNNGSNYTQKKPYNSNRPHQQRGGKFGPNRYNNRGNYNGGGSFRGGHMGANSSNVPWTGYYNNYPVYYQPCQMAAAGSAPANPIPVEEKSPVPTKIEITTKSGEHLDLKEQHKAKLQSQERSTVSPQPESKLKETSDSTSTSTPTPTPSTNDSKASSEENISEAEKTRRNFIEQVKLRKAALEKKRKEQLEGSSGNNNIPMKTTPENVEEK | | | | | |  |  |
| Recombinant eIF4G1_1-249_ S200C | This paper | | | | | | gsMTDETAHPTQSASKQESAALKQTGDDQQESQQQRGYTNYNNGSNYTQKKPYNSNRPHQQRGGKFGPNRYNNRGNYNGGGSFRGGHMGANSSNVPWTGYYNNYPVYYQPQQMAAAGSAPANPIPVEEKSPVPTKIEITTKSGEHLDLKEQHKAKLQSQERSTVSPQPESKLKETSDSTSTSTPTPTPSTNDSKASSEENICEAEKTRRNFIEQVKLRKAALEKKRKEQLEGSSGNNNIPMKTTPENVEEK | | | | | |  |  |
| Recombinant eIF4G1_1-249_ (∆Box1) | This paper | | | | | | gsMTDETAHPTQSASKQESAALKQTGDDQQESQQQRGYTNYNNGSNYTQKKPYNSNRPHQQRGGKFGPNRYNNRGNYNGGGSFRGGHMGANSSNVAAAGSAPANPIPVEEKSPVPTKIEITTKSGEHLDLKEQHKAKLQSQERSTVSPQPESKLKETSDSTSTSTPTPTPSTNDSKASSEENISEAEKTRRNFIEQVKLRKAALEKKRKEQLEGSSGNNNIPMKTTPENVEEK | | | | | |  |  |
| Recombinant eIF4G1_1-184_ | (Santiveri et al., 2011) | | | | | | gsMTDETAHPTQSASKQESAALKQTGDDQQESQQQRGYTNYNNGSNYTQKKPYNSNRPHQQRGGKFGPNRYNNRGNYNGGGSFRGGHMGANSSNVPWTGYYNNYPVYYQPQQMAAAGSAPANPIPVEEKSPVPTKIEITTKSGEHLDLKEQHKAKLQSQERSTVSPQPESKLKETSDSTSTSTPTP | | | | | |  |  |
| Recombinant eIF4G1_1-184_ W95A | This paper | | | | | | gsMTDETAHPTQSASKQESAALKQTGDDQQESQQQRGYTNYNNGSNYTQKKPYNSNRPHQQRGGKFGPNRYNNRGNYNGGGSFRGGHMGANSSNVPATGYYNNYPVYYQPQQMAAAGSAPANPIPVEEKSPVPTKIEITTKSGEHLDLKEQHKAKLQSQERSTVSPQPESKLKETSDSTSTSTPTP | | | | | |  |  |
| Recombinant eIF4G1_1-184_ Y98A/Y99A | This paper | | | | | | gsMTDETAHPTQSASKQESAALKQTGDDQQESQQQRGYTNYNNGSNYTQKKPYNSNRPHQQRGGKFGPNRYNNRGNYNGGGSFRGGHMGANSSNVPWTGAANNYPVYYQPQQMAAAGSAPANPIPVEEKSPVPTKIEITTKSGEHLDLKEQHKAKLQSQERSTVSPQPESKLKETSDSTSTSTPTP | | | | | |  |  |
| Recombinant eIF4G1_1-184_ Y105A/Y106A | This paper | | | | | | gsMTDETAHPTQSASKQESAALKQTGDDQQESQQQRGYTNYNNGSNYTQKKPYNSNRPHQQRGGKFGPNRYNNRGNYNGGGSFRGGHMGANSSNVPWTGYYNNYPVAAQPQQMAAAGSAPANPIPVEEKSPVPTKIEITTKSGEHLDLKEQHKAKLQSQERSTVSPQPESKLKETSDSTSTSTPTP | | | | | |  |  |
| Recombinant eIF4G1_1-184_ (∆Box1) | This paper | | | | | | gsMTDETAHPTQSASKQESAALKQTGDDQQESQQQRGYTNYNNGSNYTQKKPYNSNRPHQQRGGKFGPNRYNNRGNYNGGGSFRGGHMGANSSNVAAAGSAPANPIPVEEKSPVPTKIEITTKSGEHLDLKEQHKAKLQSQERSTVSPQPESKLKETSDSTSTSTPTP | | | | | |  |  |
| Recombinant eIF4G1_1-184_ (∆Box2) | This paper | | | | | | gsMTDETAHPTQSASKQESAALKQTGDDQQESQQQRGYTNYNNGSNYTQKKPYNSNRPHQQRGGKFGPNRYNNRGNYNGGGSFRGGHMGANSSNVPWTGYYNNYPVYYQPQQMAAAGSAPANPIPVEEKKAKLQSQERSTVSPQPESKLKETSDSTSTSTPTP | | | | | |  |  |
| Recombinant eIF4G1_1-82_ | (Santiveri et al., 2011) | | | | | | gsMTDETAHPTQSASKQESAALKQTGDDQQESQQQRGYTNYNNGSNYTQKKPYNSNRPHQQRGGKFGPNRYNNRGNYNGGGSFR | | | | | |  |  |
| Recombinant eIF4G1_187-234_ | This paper | | | | | | gsTNDSKASSEENISEAEKTRRNFIEQVKLRKAALEKKRKEQLEGSSG | | | | | |  |  |
| Recombinant Pub1 RRM3 | (Santiveri et al., 2011) | | | | | | gsQTIGLPPQVNPQAVDHIIRSAPPRVTTAYIGNIPHFATEADLIPLFQNFGFILDFKHYPEKGCCFIKYDTHEQAAVCIVALANFPFQGRNLRTGWGKER | | | | | |  |  |
| Recombinant protein Pab1 RRM12 | This paper | | | | | | gsMADITDKTAEQLENLNIQDDQKQAATGSESQSVENSSASLYVGDLEPSVSEAHLYDIFSPIGSVSSIRVCRDAITKTSLGYAYVNFNDHEAGRKAIEQLNYTPIKGRLCRIMWSQRDPSLRKKGSGNIFIKNLHPDIDNKALYDTFSVFGDILSSKIATDENGKSKGFGFVHFEEEGAAKEAIDALNGMLLNGQEIYVAPHLSRKERDSQLE | | | | | |  |  |
| Protein eIF4G1_35-49_-Pub1 RRM3 | This paper | | | | | | | gsGYTNYNNGSNYTQKKQTIGLPPQVNPQAVDHIIRSAPPRVTTAYIGNIPHFATEADLIPLFQNFGFILDFKHYPEKGCCFIKYDTHEQAAVCIVALANFPFQGRNLRTGWGKER | | | | |  |  |
| Protein Pub1 RRM3-eIF4G1_35-49_ | This paper | | | | | | | gsQTIGLPPQVNPQAVDHIIRSAPPRVTTAYIGNIPHFATEADLIPLFQNFGFILDFKHYPEKGCCFIKYDTHEQAAVCIVALANFPFQGRNLRTGWGKERGYTNYNNGSNYTQKK | | | | |  |  |
| Software and Algorithms | | | | | | | | | | | | |  |  |
| TopSpin 4.1 | | Bruker | | | | | | | | | https://www.bruker.com | |  |  |
| nmrPipe | | IBBS | | | | | | | | | https://www.ibbr.umd.edu/nmrpipe/index.html | |  |  |
| TALOS+ | | (Shen et al., 2009) | | | | | | | | |  | |  |  |
| PALES | | (Zweckstetter and Bax, 2000) | | | | | | | | |  | |  |  |
| ASTRA SEC-software version 5.3.4 | | Wyatt Technology | | | | | | | | | https://www.wyatt.com | |  |  |
| Dynamics V6 | | Wyatt Technology | | | | | | | | | https://www.wyatt.com | |  |  |
| CcpNmr Analysis | | (Vranken et al., 2005) | | | | | | | | | https://www.ccpn.ac.uk | |  |  |
| Camshift | | (Camilloni et al., 2012) | | | | | | | | |  | |  |  |
| Cyana | | (Guntert and Buchner, 2015) | | | | | | | | | V3.98.13 | |  |  |
| Flexible Meccano | | (Ozenne et al., 2012) | | | | | | | | | V1.1 | |  |  |
| Pymol | | Schödinger | | | | | | | | | https://pymol.org/ | |  |  |
| ApE | | Plasmid Editor by M. Wayne Davis | | | | | | | | | jorgensen.biology.utah.edu/wayned/ape/ | |  |  |
| ATSAS | | (Franke et al., 2017) | | | | | | | | | V 3.0.0.3 | |  |  |
| CRYSOL | | (Svergun et al., 1995) | | | | | | | | | N/A | |  |  |
| EOM | | (Bernado et al., 2007) | | | | | | | | | N/A | |  |  |
| Other | | | | | | | | | | | | |  |  |
| Vivaspin 20, 5kDa MWCO concentrators | | | Sigma Aldrich | | | | | | | | Z614580 | |  |  |
| Vivaspin 15, 10kDa MWCO concentrators | | | Sigma Aldrich | | | | | | | | VS15T01 | |  |  |
| Vivaspin 6, 10kDa MWCO concentrators | | | Sigma Aldrich | | | | | | | | Z614467 | |  |  |
| Hitrap^TM^ IMAC FF | | | GE Healthcare | | | | | | | | 17-0921-04 | |  |  |
| Hitrap^TM^ Q HP | | | GE Healthcare | | | | | | | | 17-1154-01 | |  |  |
| Hitrap^TM^ SP HP | | | GE Healthcare | | | | | | | | 17-1152-01 | |  |  |
| Bio-Scale^TM^ bio-gel P-6 Desalting Cartridge | | | BIO-RAD | | | | | | | | 732-5312 | |  |  |
| Nap^TM^ -5 Columns Sephadex^TM^ G-25 DNA Grade | | | GE Healthcare | | | | | | | | 17-0853-01 | |  |  |
| Superdex^TM^ 200 10/300 GL | | | GE Healthcare | | | | | | | | 17-5157-01 | |  |  |
